# Exploring the molecular basis of neuronal excitability in a vocal learner

**DOI:** 10.1186/s12864-019-5871-2

**Published:** 2019-08-02

**Authors:** Samantha R. Friedrich, Peter V. Lovell, Taylor M. Kaser, Claudio V. Mello

**Affiliations:** Department of Behavioral Neuroscience, Oregon Health and Science University, 3181 Sam Jackson Park Rd L470, Portland, OR USA

**Keywords:** Birdsong, Vocal learning, Zebra finch, Neuronal excitability, Ion channels, Gene expression, Action potential, Comparative genomics

## Abstract

**Background:**

Vocal learning, the ability to learn to produce vocalizations through imitation, relies on specialized brain circuitry known in songbirds as the song system. While the connectivity and various physiological properties of this system have been characterized, the molecular genetic basis of neuronal excitability in song nuclei remains understudied. We have focused our efforts on examining voltage-gated ion channels to gain insight into electrophysiological and functional features of vocal nuclei. A previous investigation of potassium channel genes in zebra finches (*Taeniopygia guttata*) revealed evolutionary modifications unique to songbirds, as well as transcriptional specializations in the song system [Lovell PV, Carleton JB, Mello CV. BMC Genomics 14:470 2013]. Here, we expand this approach to sodium, calcium, and chloride channels along with their modulatory subunits using comparative genomics and gene expression analysis encompassing microarrays and in situ hybridization.

**Results:**

We found 23 sodium, 38 calcium, and 33 chloride channel genes (HGNC-based classification) in the zebra finch genome, several of which were previously unannotated. We determined 15 genes are missing relative to mammals, including several genes (CLCAs, BEST2) linked to olfactory transduction. The majority of sodium and calcium but few chloride channels showed differential expression in the song system, among them SCN8A and CACNA1E in the direct motor pathway, and CACNG4 and RYR2 in the anterior forebrain pathway. In several cases, we noted a seemingly coordinated pattern across multiple nuclei (SCN1B, SCN3B, SCN4B, CACNB4) or sparse expression (SCN1A, CACNG5, CACNA1B).

**Conclusion:**

The gene families examined are highly conserved between avian and mammalian lineages. Several cases of differential expression likely support high-frequency and burst firing in specific song nuclei, whereas cases of sparse patterns of expression may contribute to the unique electrophysiological signatures of distinct cell populations. These observations lay the groundwork for manipulations to determine how ion channels contribute to the neuronal excitability properties of vocal learning systems.

**Electronic supplementary material:**

The online version of this article (10.1186/s12864-019-5871-2) contains supplementary material, which is available to authorized users.

## Background

Motor learning, the process by which motor skills are acquired and perfected through practice, requires fine-tuning of sensorimotor circuit elements to ultimately produce precise motor output. One remarkable example is vocal learning, a trait that allows individuals to learn their vocalizations through auditorily guided vocal practice. Vocal learning is demonstrated only by some mammals (humans, cetaceans, bats, and possibly pinnipeds and elephants), and three groups of birds (parrots, hummingbirds, and songbirds) [1]. Songbirds provide a particularly powerful model for studying the role of specific genes within vocal learning circuits. Decades of study in the zebra finch have revealed much about neuroanatomical substrates for vocal learning, including the connectivity and electrophysiological properties of a discrete set of vocal nuclei called the song system [2, 3]. Single-unit neural recordings and modeling have described the electrophysiological characteristics and underlying conductances of several song system neuron types [4–13]. Less established is how the regulated expression of genes - specifically ion channel genes - gives rise to the physiological and excitable properties of song system neurons.

**Fig. 1:**
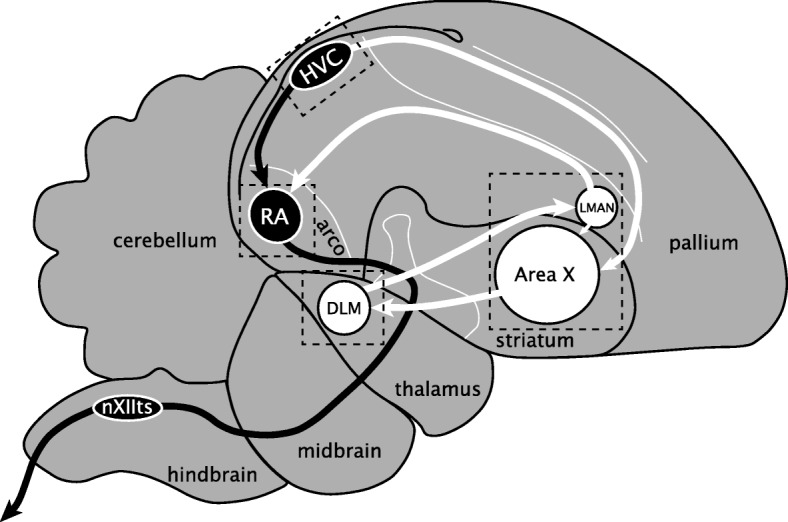
The zebra finch song system. Diagram of major brain areas collapsed across parasagittal planes to show the approximate locations and connections of the song control nuclei (not all connections are shown). The direct motor pathway (DMP; black arrows and nuclei) encompasses the projection from HVC to RA and from RA to vocal-motor nucleus nXIIts, the output of which controls the syrinx. The anterior forebrain pathway (AFP; white arrows and nuclei) encompasses the projection from HVC to Area X, from Area X to DLM, from DLM to LMAN, from LMAN back to Area X, and from LMAN to RA. Dotted boxes indicate approximate positions of the in situ photomicrographs presented in Figs. 7 8, 9, 10, 11. See text for anatomical abbreviations

The zebra finch song system is composed of the posterior direct motor pathway (DMP), necessary for song production [14, 15], and the anterior forebrain pathway (AFP) necessary for song learning and adult song variability [16–19]. Nucleus HVC (proper name) projects to both the DMP and AFP (Fig. 1), and receives inputs from nucleus interfacialis of the nidopallium (NIf) [20] and thalamic nucleus uvaeformis (Uva) [21]. In the DMP, terminals from HVC synapse onto neurons of the robust nucleus of the arcopallium (RA), which are considered analogous to layer 5/6 motor neurons of mammalian laryngeal motor cortex [22]. RA then projects to the midbrain’s dorsomedial (DM) nucleus of the intercollicular complex, to the tracheosyringeal subdivision of the XII cranial nerve nucleus (nXIIts), which innervates the syrinx (the avian vocal organ), and to medullary respiratory centers [23]. In the AFP, striatal Area X receives projections from HVC and projects to the medial nucleus of dorsolateral thalamus (DLM), which projects to the lateral magnocellular nucleus of the anterior nidopallium (LMAN) [24]. LMAN projects to Area X [25] but also to RA, converging with inputs from the DMP [26]. Detailed knowledge of this circuitry facilitates the identification of the molecular genetic determinants of its electrophysiological properties.

Given their direct effects on membrane potential, cell excitability, and neuronal firing, ion channels genes are among the most important determinants of neuronal function [27–29]. Their regulation and modulation is important in establishing electrophysiological properties of specific circuits, and may also contribute to nonsynaptic mechanisms of learning and memory [30–32]. As such, much can be learned about circuit function and excitability properties by identifying regional differences in ion channel gene expression. We previously examined the genomics and brain expression of potassium channel genes in zebra finch [33] and uncovered evidence of differential expression for several key regulators of resting membrane and action potential repolarization. Here, we extend our analysis to sodium, calcium, and chloride channel families, all containing genes involved in determining neuronal excitability properties. Whereas sodium currents drive the rising phase of the action potential (AP) to ensure its initiation and propagation, calcium channels perform a wide array of functions such as regulating synaptic vesicle release, activating second messenger systems, and integrating signals over dendrites [27, 34, 35]. While less is known about chloride channels, some members of this family play a role in stabilizing resting membrane potential [36].

We identified 23 sodium, 38 calcium, and 33 chloride channel genes in the zebra finch genome - the majority with clear orthologs in mammals, indicating their broad conservation across vertebrates. We also established complements of ion channel genes that are differentially expressed within the major song system nuclei compared to the surround. These data represent the most comprehensive characterization of these ion channel gene families in a bird species to date. They also provide novel insights into how molecular specializations associated with neuronal excitability may have evolved to support a complex, learned vocal behavior.

## Methods

### Determining the full complement of sodium, calcium, and chloride channel genes in the zebra finch genome

We identified the sets of sodium, calcium, and chloride channel genes in zebra finch using a modified strategy of that previously used for potassium channels [33]. We first compiled the full set of human orthologs for these genes by navigating gene family hierarchies in HGCN (Ion channels > Ion channels by channel type > Sodium channels / Calcium channels / Chloride channels) and retrieving all gene names within all subfamilies for each of these three ion channel families [37]. Starting with human, arguably one of the most complete and best annotated genomes, allowed us to define a comprehensive set of genes to be examined. This effort identified a total of 108 ion channel genes, which included 23 sodium, 43 calcium, and 41 chloride channel and channel-related genes. This comprehensive set included genes encoding ion channels which have been linked to neuronal transmission, as well as the auxiliary subunits that modulate these channels. It also includes epithelial sodium channel (SCNN) genes, sperm-associated cation channel (CATSPER) genes, and volume-regulated anion channel subunits (LRRC8), even though these families have not been implicated in neuronal excitability and their expression in mammals is generally biased toward non-neuronal tissues [38–40].

We next searched for the orthologous genes in zebra finch using the following pipeline: (1) Obtain from NCBI (National Center for Biotechnology Information) the human records for the HGNC (HUGO Gene Nomenclature Committee) gene list; (2) Align the human models to the zebra finch genome to identify potential orthologous loci; (3) Verify the synteny of the top-scoring alignment; 4) Demonstrate that the zebra finch gene aligns preferentially to the corresponding human locus; and (5) Demonstrate that secondary alignments represent other family members. These steps were also applied to identify chicken orthologs (“Chicken locus” column of Additional file 1: Table S1) in the galGal5 (GCA_000002315.3 [41]) or updated galGal6 (GRCg6a; GCA_000002315.5) chicken genome. Each step is detailed next and graphically summarized in Additional file 2.

(1) We queried the NCBI Entrez Gene database with the names of the 108 genes in the starting HGNC gene list, to retrieve the corresponding human gene records. All 108 human genes were found in NCBI. (2) We retrieved the human RefSeq nucleotide sequences of the longest isoform for each annotated gene and used these as queries to search the zebra finch genome (taeGut1/WUGSC 3.2.4; GCA_000151805; Jul 2008 [42]) using the UCSC Browser BLAT [43], noting all significant hits, defined as BLAT scores above 50 (top BLAT scores for human-to-taeGut1 alignments are reported in Additional file 1: Table S1). (3) We compared the synteny of the top scoring alignment in finch with that of the human query. We manually examined the immediately flanking genes on both sides of the alignment and query, if necessary expanding our analysis to genes farther up- or downstream. (4) The gene prediction (Ensembl release 95 [44]) at the top scoring finch locus was aligned back to human (using BLAT and/or BLAST) and the top-scoring alignment in human examined to confirm it was to the expected locus (same as initial query). For zebra finch gene models with multiple transcripts in Ensembl, we selected the longest variant. (5) All significant secondary alignments found in the finch genome were verified as done for the top hits (synteny comparison and back-alignment to human). This step served both to further confirm the orthology of the top hit and to identify possible novel paralogs or paralogs missing from the human gene list (e.g. TPCN3) in the zebra finch genome.

In the majority of cases, the top-scoring alignments in both finch and humans were reciprocally aligning models with conserved synteny across species, and all significant secondary alignments were to other genes of the same family, thus confirming orthology to human of the primary alignment in finch. These zebra finch Ensembl models that passed our synteny and alignment criteria are reported in Table 1 (*n* = 61 genes, no symbols next to gene name and the “Zebra finch locus” column contains only Ensembl model(s)). We detected no cases of multiple top-scoring alignments with similar or different syntenies, thus we conclude that no novel paralogs and/or segmental duplications (e.g. of a cluster of syntenic genes) are present in the zebra finch genome for the gene families examined. In a few cases no Ensembl model was present but synteny of the top scoring finch locus was conserved with the human ortholog (*n* = 2; chromosome location in taeGut1 is indicated in the “Zebra finch locus” column of Table 1). Lastly, secondary high scoring BLAT alignments to chrUn (chromosome Unknown) were not examined further, as those cases represent allelic variants in the zebra finch genome (as detailed in [42]).

**Table 1:** Ion channel genes in the zebra finch genome

HGNC Symbol	Alternate name	Full name	Zebra finch locus
**A. Sodium channels**			
*α subunits*			
SCN1A	Nav1.1	sodium voltage-gated channel alpha subunit 1	ENSTGUG00000007356^
SCN2A	Nav1.2	sodium voltage-gated channel alpha subunit 2	ENSTGUG00000007152^*
SCN3A	Nav1.3	sodium voltage-gated channel alpha subunit 3	ENSTGUG00000007034
SCN4A	Nav1.4	sodium voltage-gated channel alpha subunit 4	ENSTGUG00000003230
SCN5A	Nav1.5	sodium voltage-gated channel alpha subunit 5	ENSTGUG00000000531^
			ENSTGUG00000000533^
SCN8A	Nav1.6	sodium voltage-gated channel alpha subunit 8	ENSTGUG00000003307
SCN9A	Nav1.7	sodium voltage-gated channel alpha subunit 9	ENSTGUG00000007470^*
SCN10A	Nav1.8	sodium voltage-gated channel alpha subunit 10	ENSTGUG00000000476^*
SCN11A	Nav1.9	sodium voltage-gated channel alpha subunit 11	chr2:5686797–5,719,627
*β subunits*			
SCN1B^$^		sodium voltage-gated channel beta subunit 1	MUGN01000920.1818309–818,447
SCN2B		sodium voltage-gated channel beta subunit 2	ENSTGUG00000017358
			MUGN01000074.12766152–2,766,960
SCN3B		sodium voltage-gated channel beta subunit 3	ENSTGUG00000000607^
SCN4B		sodium voltage-gated channel beta subunit 4	ENSTGUG00000000230
			MUGN01000074.12777132–2,778,696
*Acid-sensing*			
ASIC1	ACCN2	acid sensing ion channel subunit 1	ENSTGUG00000003637
ASIC2^#^	ACCN1	acid sensing ion channel subunit 2	ENSTGUG00000003212^*
			ENSTGUG00000003215^*
ASIC3^◆^	ACCN3	acid sensing ion channel subunit 3	ENSTGUG00000016020^*
			MUGN01000217.1876923–883,599
ASIC4	ACCN4	acid sensing ion channel subunit 4	ENSTGUG00000006157*
ASIC5^◆^	ACCN5	acid sensing ion channel subunit 5	ENSTGUG00000005392
*Leak channel*			
NALCN		sodium leak channel, non selective	ENSTGUG00000010876
*Epithelial*			
SCNN1A^◆$^	SCNN1	sodium channel epithelial 1 alpha subunit	ENSTGUG00000013342*
SCNN1B		sodium channel epithelial 1 beta subunit	ENSTGUG00000005936
SCNN1D^◆^		sodium channel epithelial 1 delta subunit	ENSTGUG00000004091
SCNN1G		sodium channel epithelial 1 gamma subunit	chr14:8747853–8,755,433
**B. Calcium channels**			
*L-type α subunits*			
CACNA1S	Cav1.1	calcium voltage-gated channel subunit alpha1 S	ENSTGUG00000001142*
CACNA1C	Cav1.2	calcium voltage-gated channel subunit alpha1 C	ENSTGUG00000012529^
			ENSTGUG00000012538^
CACNA1D	Cav1.3	calcium voltage-gated channel subunit alpha1 D	ENSTGUG00000006839*
CACNA1F	Cav1.4	calcium voltage-gated channel subunit alpha1 F	MUGN01000244.140490–59,194
*N/P/Q/R- type α subunits*			
CACNA1A	Cav2.1	calcium voltage-gated channel subunit alpha1 A	MUGN01001147.1274884–306,034
CACNA1B	Cav2.2	calcium voltage-gated channel subunit alpha1 B	ENSTGUG00000002855*
CACNA1E	Cav2.3	calcium voltage-gated channel subunit alpha1 E	ENSTGUG00000017352
*T-type α subunits*			
CACNA1G	Cav3.1	calcium voltage-gated channel subunit alpha1 G	ENSTGUG00000009049*
CACNA1H	Cav3.2	calcium voltage-gated channel subunit alpha1 H	ENSTGUG00000006881*
CACNA1I	Cav3.3	calcium voltage-gated channel subunit alpha1 I	ENSTGUG00000010198*
*α2/δ subunits*			
CACNA2D1		calcium voltage-gated channel auxiliary subunit alpha2delta 1	ENSTGUG00000002536^*
			ENSTGUG00000002533^*
CACNA2D2		calcium voltage-gated channel auxiliary subunit alpha2delta 2	ENSTGUG00000004703^*
			ENSTGUG00000004711^*
			ENSTGUG00000004714^*
CACNA2D3		calcium voltage-gated channel auxiliary subunit alpha2delta 3	ENSTGUG00000006966^*
CACNA2D4		calcium voltage-gated channel auxiliary subunit alpha2delta 4	ENSTGUG00000012579^
*β subunits*			
CACNB1		calcium voltage-gated channel auxiliary subunit beta 1	MUGN01000261.12481145–2,492,038
CACNB2		calcium voltage-gated channel auxiliary subunit beta 2	ENSTGUG00000001247*
CACNB3		calcium voltage-gated channel auxiliary subunit beta 3	ENSTGUG00000015546
			MUGN01000394.1996686–999,415
CACNB4		calcium voltage-gated channel auxiliary subunit beta 4	ENSTGUG00000012082^*
*γ subunits*			
CACNG1		calcium voltage-gated channel auxiliary subunit gamma 1	ENSTGUG00000004397^
CACNG2	stargazer,	calcium voltage-gated channel auxiliary subunit gamma 2	ENSTGUG00000010725
	stargazin		
CACNG3		calcium voltage-gated channel auxiliary subunit gamma 3	ENSTGUG00000006208*
CACNG4		calcium voltage-gated channel auxiliary subunit gamma 4	ENSTGUG00000004385*
CACNG5		calcium voltage-gated channel auxiliary subunit gamma 5	ENSTGUG00000004375*
CACNG7^$^		calcium voltage-gated channel auxiliary subunit gamma 7	MUGN01000421.1339861–356,707
CACNG8^$^		calcium voltage-gated channel auxiliary subunit gamma 8	MUGN01000421.1325281–333,090
*Intracellular*			
ITPR1	IP3R1	inositol 1,4,5-trisphosphate receptor type 1	ENSTGUG00000010241^
ITPR2	IP3R2	inositol 1,4,5-trisphosphate receptor type 2	ENSTGUG00000012196^
ITPR3	IP3R3	inositol 1,4,5-trisphosphate receptor type 3	ENSTGUG00000001798
RYR1		ryanodine receptor 1	ENSTGUG00000016333^
			MUGN01000615.12737–83,931
RYR2		ryanodine receptor 2	ENSTGUG00000010491
RYR3		ryanodine receptor 3	ENSTGUG00000011653^
TPCN1	TPC1	two pore segment channel 1	ENSTGUG00000009086
TPCN2^◆^	TPC2	two pore segment channel 2	ENSTGUG00000005328
TPCN3^◆✣^	TPC3	two pore segment channel 3	ENSTGUG00000007338^*
			ENSTGUG00000015038^*
*Sperm associated*			
CATSPERB^▽◆^	C14orf161	cation channel sperm associated auxiliary subunit beta	MUGN01001095.110976025–10,976,136
CATSPERD^▽◆$^	TMEM146	cation channel sperm associated auxiliary subunit delta	MUGN01000898.110200480–10,200,746
CATSPERE^▽◆^	C1orf101	catsper channel auxiliary subunit epsilon	ENSTGUG00000008288^*
			MUGN01000667.120007852–20,020,082
CATSPER3^▽◆^		cation channel sperm associated 3	chr13_random:2493868–2,500,983
			MUGN01000154.1512621–517,332
**C. Chloride channels**			
*CLCNs*			
CLCN1	CLC1	chloride voltage-gated channel 1	ENSTGUG00000013222*
CLCN2	CLC2	chloride voltage-gated channel 2	ENSTGUG00000010323*
CLCN3	CLC3	chloride voltage-gated channel 3	ENSTGUG00000006161
CLCN4	CLC4	chloride voltage-gated channel 4	ENSTGUG00000008365
CLCN5^$^	CLC5	chloride voltage-gated channel 5	ENSTGUG00000005324
CLCN6	CLC6	chloride voltage-gated channel 6	ENSTGUG00000002366*
CLCN7	CLC7	chloride voltage-gated channel 7	ENSTGUG00000004211*
CLCNK^✝^	CLCK1	chloride voltage-gated channel K^✝^	ENSTGUG00000002023^*
BSND	BART, DFNB73	barttin CLCNK type accessory beta subunit A	chr8:22970234:22970413
*CLICs*			
CLIC2^#^	CLIC2b	chloride intracellular channel 2	ENSTGUG00000004925
CLIC3^$^		chloride intracellular channel 3	ENSTGUG00000002341*
CLIC4	CLIC4L	chloride intracellular channel 4	ENSTGUG00000001132^*
CLIC5		chloride intracellular channel 5	ENSTGUG00000013246
CLIC6^◆^	CLIC1L	chloride intracellular channel 6	ENSTGUG00000004696
			MUGN01000638.1213150–238,240
CLCC1^◆^	MCLC	chloride channel CLIC like 1	ENSTGUG00000004508*
*Calcium-activated*			
ANO1	TMEM16A	anoctamin 1	ENSTGUG00000005385*
ANO2	TMEM16B	anoctamin 2	ENSTGUG00000011938
ANO3	TMEM16C	anoctamin 3	ENSTGUG00000004669
ANO4	TMEM16D	anoctamin 4	ENSTGUG00000009008
ANO5	TMEM16E	anoctamin 5	ENSTGUG00000004532*
ANO6	TMEM16F	anoctamin 6	ENSTGUG00000006024*
ANO7^▽^	TMEM16G	anoctamin 8	MUGN01000068.1571318–573,272
ANO8	TMEM16H	anoctamin 8	ENSTGUG00000015909^*
ANO9	TMEM16J	anoctamin 9	ENSTGUG00000006753
ANO10	TMEM16K	anoctamin 10	ENSTGUG00000003830
BEST1^◆^	VMD2	bestrophin 1	ENSTGUG00000005934^*
BEST3	VMD2L3	bestrophin 3	ENSTGUG00000006968^
*Volume regulated*			
LRRC8A	LRRC8	leucine rich repeat containing 8 VRAC subunit A	ENSTGUG00000004233
LRRC8B		leucine rich repeat containing 8 VRAC subunit B	ENSTGUG00000006230*
LRRC8C		leucine rich repeat containing 8 VRAC subunit C	ENSTGUG00000006226
LRRC8D	LRRC5	leucine rich repeat containing 8 VRAC subunit D	ENSTGUG00000006223
*Other*			
CFTR		cystic fibrosis transmembrane conductance regulator	ENSTGUG00000004828
CLNS1A^◆^		chloride nucleotide-sensitive channel 1A	ENSTGUG00000013030^

^▽^ Highly partial or truncated

^✝^Suggested name (not clear whether this locus is orthologous to mammalian CLCNKA or CLCNKB)

^$^ Synteny traced through phylogenetic relationships

^#^ Synteny could not be linked through phylogenetic relationship

^◆^ Non-human ortholog used to find locus

^✣^ No human ortholog

* Less than 90% of post-recovery length

^ Annotated as a “novel gene” in Ensembl

Several groups of genes required further verification efforts and/or variations of the main pipeline described above; these cases are discussed next and graphically summarized in Additional file 2.

#### Case 1: Gene clusters

Some sodium channel (SCNxA) genes are arranged in clusters; in such cases, BLAT alignments tend to span multiple family members, or identify another family member in a given cluster. For these genes, we compared the syntenies within and around the clusters as a group across species, and closely examined the number, orientation and alignments of the individual genes in each cluster before deciding orthology. Both clusters in this family were found conserved between finch and humans (*n* = 7 genes in 2 clusters: SCN5A, SCN10A, SCN11A, and SCN1A, SCN2A, SCN3A, SCN9A).

#### Case 2: Gene in taeGut1 but synteny diverges from human

**Fig. 2:**
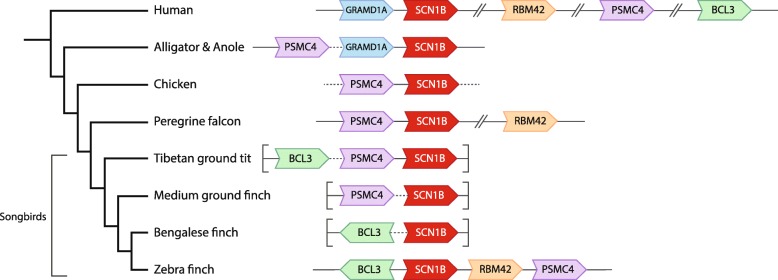
SCN1B is present in birds. SCN1B (red) is missing from taeGut1 but present in the zebra finch PacBio assembly. The different orientations (arrows point in 5′ to 3′ direction) and relative positions of BCL3, PSMC4, and RBM42 across birds suggest multiple rearrangement events in the avian phylogeny, even within different songbird lineages. GRAMD1A was not found in any avian species. Arrowheads indicate gene orientation; solid lines indicate gap-less intergenic regions; dotted lines indicate intergenic regions containing gaps; broken lines indicate interspersed genes not shown; brackets indicate the ends of scaffolds in the respective assemblies

**Fig. 3:**
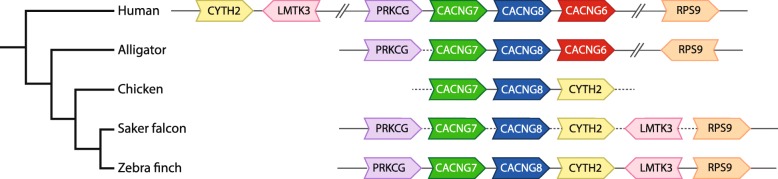
CACNG6 is missing from birds. While CACNG7 and CACNG8 are present, the syntenic paralog CACNG6 cannot be found in birds. The different orientations (arrows point in 5′ to 3′ direction) and relative positions of CYTH2, LMTK3, and RPS9 across phylogeny suggests multiple rearrangement events in this genomic region, possibly in an avian ancestor. Arrowheads indicate gene orientation; solid lines indicate gap-less intergenic regions; dotted lines indicate intergenic regions containing gaps; broken lines indicate interspersed genes not shown

Some genes met the reciprocal top-scoring alignment criteria but their synteny did not match that of human. To examine them further, we compared the synteny in zebra finch and human to that of orthologous loci in other birds, including Bengalese finch, medium ground finch, and tit (other passeriforms), falcon (outgroup to psitacopasseridae), chicken and/or quail (galliformes, basal neoaves), tinamou, ostrich, and emu (ratites, basal avian), lizard, alligator and turtle (non-avian sauropsids, outgroup to birds), Xenopus (amphibian, outgroup to amniotes), and platypus and opossum (basal mammals). We only used non-human orthologs that met two criteria: (1) conserved synteny and (2) aligned preferentially to the correct human locus. For 4 genes, this phylogenetic approach revealed an indirect relationship between the syntenies of the human query and the top scoring alignment in finch (indicated by a “$” in Table 1; details in “Synteny notes” column of Additional file 1: Table S1; examples in Figs. 2 and 3). For another 2 genes (ASIC2 and CLIC2), human and zebra finch syntenies could not be fully linked through phylogenetic relationships, indicating weaker evidence of orthology (indicated by a “#” in Table 1).

#### Case 3: Gene detection in taeGut1 required non-human ortholog queries

In other cases, there were no significant alignments of the human query to taeGut1. For these genes, we pursued further searches in taeGut1 using as queries orthologs from the same list of other species discussed above in Case 2. For all 6 genes in this category, the top-scoring alignment identified a zebra finch Ensembl model that itself aligned to the correct locus in human, and whose synteny was conserved with human (indicated by a “◆” in Table 1 and a chromosome location in the “Zebra finch locus” column). In the case of TPCN3, which lacks a human model, we used orthologs from other species (chicken, dog) as queries to confirm the locus (initially identified from secondary alignments of human TPCN1 and 2) in taeGut1, and confirmed that synteny in zebra finch was conserved with those species.

#### Case 4: Synteny verification required examination of PacBio assembly

In yet other cases, a locus with an Ensembl model in taeGut1 was identified through alignments of human or other species’ models and that Ensembl model aligned back to the correct locus in human but lacked syntenic context. This occurred when the top-scoring alignment in finch was located between gaps and/or on an unplaced scaffold or a chromosome “random” (e.g. chr13_random), or when the only significant alignment was to chrUn. For these genes, we next used the zebra finch Ensembl model to conduct searches of a newer zebra finch assembly (Tgut_diploid_1.0; GCA_002008985; Korlach et al. 2017) built exclusively from PacBio reads (Pacific Biosciences). This PacBio assembly has higher contiguity, fewer gaps (individual scaffolds have no gaps), and often more genes and intergenic regions than the Sanger, 454, or Illumina assemblies [45]. Since this PacBio assembly currently does not have gene predictions, the PacBio scaffolds found to contain the top scoring alignments were examined further by BLASTing against RefSeq databases [46]. Briefly, we BLAST searched the NCBI collections of avian and non-avian RefSeqs separately, using as query the identified PacBio scaffolds, broken down into 10–40 kb segments depending on gene size and gene density. The ion channel gene of interest was confirmed as present in zebra finch if the BLAST alignment output had high-scoring alignments of: a) the correct orthologs from multiple non-avian species at the same locus, and b) neighboring genes to that locus that confirmed a conserved synteny with human or other species as detailed in Case 2. All 8 genes in this category are indicated by the presence of both an Ensembl model and a PacBio location in the “Zebra finch locus” column of Table 1). We note that in 2 of these 8 cases (ASIC3 and CATSPERE), the top scoring alignment back to human was not to the correct locus, but both Ensembl models are very partial relative to their full-length human ortholog. In such cases the query seems to be targeting a conserved region or motif rather than the entire gene, thus the reciprocal alignment is not an accurate criterion.

#### Case 5: Gene located only in PacBio

In several other cases, no locus could be found through alignments of human or other species’ orthologs to taeGut1. For these genes, we searched the PacBio assembly with the human model and/or other species’ orthologs and confirmed synteny using BLAST to explore the surrounding scaffold sequence (see example in Additional file 3), as described in Cases 3 and 4 above. As there are no models in the PacBio assembly, we did not perform alignments of the zebra finch locus to human. In all cases in this category, we found a correct ortholog (*n* = 10; genes have a PacBio location instead of an Ensembl model in the “Zebra finch locus” column of Table 1). In 5 of these 10 cases (indicated by “▽” in Table 1 and Additional file 1: Table S1), we found only short segments of the ion channel gene between its syntenic genes, indicating severe truncation. Because these PacBio scaffolds have no internal missing sequence due to gaps, such truncations point to likely examples of pseudogenization.

#### Case 6: Gene could not be found in either zebra finch assembly

When no significant hits could be recovered from alignments of orthologs in either taeGut1 or the zebra finch PacBio assembly, we followed a pipeline for candidate missing genes, specifically: (1) We BLAST searched the finch PacBio assembly using models of syntenic genes from comparative species as queries. When the syntenic genes were found on the same scaffold, we conducted additional BLAST searches of short segments (1–2 kb) of their intergenic region at a time. We interpret cases in which syntenic genes were adjacent but no traces of the ion channel gene could be found intergenically as high likelihood cases of gene loss, noting again that finch PacBio scaffolds are gapless. (2) To better understand the origin of such losses, we systematically searched for the gene of interest and syntenic genes in all birds with an assembled genome deposited in NCBI, initially based on gene name searches in Entrez Gene. In cases where other avian species’ scaffolds contained syntenic genes but lacked a model for the gene of interest, we conducted BLAST searches of the intergenic region as described in Case 4. In cases where we could not find evidence for the gene in any bird species, we applied the same general strategy above to search for the gene of interest and its syntenic context in representative outgroups (e.g. alligator, lizard), to trace the most likely pattern of gene loss in groups ancestral to birds (for example, see Fig. 3). We note, however, that there are often gaps in the intergenic regions of these Illumina-based assemblies, which limits the certainty of conclusions regarding gene loss in species currently lacking a PacBio assembly.

In all cases where the Ensembl model at the zebra finch locus was annotated, that annotation was correct. In cases where an Ensembl model was present but annotated as a “novel gene”, we annotated it accordingly (*n* = 34 models; indicated by a ‘^’ next to the Ensembl model in Table 1).

### Assessing gene model completeness and expanding gene models with additional sequence

While inspecting taeGut1 loci to verify orthology, we found many Ensembl predictions that seemed very partial. As a first pass to estimate gene model quality and completeness, we calculated a length ratio by dividing the length in bases of each zebra finch model by that of the orthologous human model (values in Additional file 1*:* Table S1; frequency histogram in Additional file 4A). As with the orthology analysis, we selected the longest transcript variant for zebra finch and human. If there were multiple non-overlapping zebra finch Ensembl models at a locus (i.e. split models), their lengths were summed.

We also used an alignment-based method to evaluate the completeness of zebra finch models. Because chicken (galGal6) had a higher coverage (82x) and contig N50 (17,655,422) than taeGut1 (5.5x; 38,639) and thus had more reliable and complete gene predictions, we used chicken models from Ensembl (release 95). RYR1 was excluded from this analysis as it is > 100 kb long, fragmented into at least 8 models across 8 different scaffolds that lack syntenic context in galGal5, and the only verified model in taeGut1 is highly partial and in a gappy region of chrUn. For all other genes with orthology-verified models in both zebra finch and chicken (n = 79 genes, models in both “Zebra finch locus” and “Chicken locus” columns of Additional file 1*:* Table S1), we first obtained the transcript sequences of the orthologous chicken Ensembl models. As with zebra finch, we selected the longest transcript variant and concatenated split models. We then aligned the chicken and corresponding zebra finch models to taeGut1 using the UCSC Browser BLAT and exported the results as psl files. Using a custom Python (version 3.6.7) script [47], we compared the top-scoring alignments of chicken and zebra finch models for each locus to find blocks of aligned chicken model sequence that did not overlap with any blocks of aligned zebra finch model sequence. These blocks of aligned chicken sequence missing from the zebra finch Ensembl gene predictions highlight the incompleteness of the zebra finch models and define sequences that can be used to expand these incomplete models (Additional file 5). We created a taeGut1 BED track that displays these additional sequence blocks in the UCSC genome browser (Additional file 6). For each gene in this analysis, we calculated the number of bases recovered, as well as a “percent of post-recovery length” (original model length / [original model length + number of bases recovered]; values in Additional file 1*:* Table S1; frequency histogram in Additional file 4B). Genes were considered problematic if the original Ensembl model length was less than 90% of the percent post-recovery length (indicated by a “*” in the “Zebra finch locus” column of Table 1, and values reported as % post-recovery length < 90% in Additional file 1*:* Table S1).

### Animal subjects and brain tissue

Adult male zebra finches were obtained from a commercial supplier (Magnolia Bird Farm, Pasadena, CA) and acclimated in our animal facility in same-sex group cages for at least 2 weeks prior to the onset of experiments. The work described in this study was approved by the OHSU IACUC and is in accordance with NIH guidelines. To minimize the confound of song-induced gene expression, all birds were placed overnight in acoustic isolation chambers and monitored for singing for a period of 2 h after lights-on. Verified non-singing birds were sacrificed by decapitation and brains were rapidly dissected, blocked in TissueTec OCT (Sakura Finetek USA, Inc.; Torrance, CA), then flash-frozen in a slurry of isopropanol and crushed dry ice. Frozen brains were sectioned in the sagittal plane on a Leica CM1850 cryostat at 10 μm and melted onto charged microscope slides (Colorfrost Plus; Thermo Fisher Scientific; Waltham, MA). Slides were stored at − 80 °C until use.

### cDNA clone selection and riboprobe synthesis

All probes were derived from clones selected from the ESTIMA zebra finch cDNA library [48]. All genes present in taeGut1 were examined in the UCSC Genome Browser for the presence of ESTIMA clones. For genes found only in the PacBio assembly, we BLASTed each corresponding scaffold region against all NCBI zebra finch EST collections to evaluate potential evidence of expression and identify suitable cDNA clones (high alignment scores and alignments that mirrored the exon structure of aligned orthologs). Preference was given to clones representing the 3’UTR region, to minimize cross-alignment of conserved coding sequences with other gene family members. Candidate ESTs were aligned to the zebra finch genome using BLAT (UCSC Genome Browser) to confirm mapped unambiguous mapping to the target gene without significant hits to other loci. Clone IDs are provided for all genes for which an in situ was run (“EST evidence” column of Additional file 1: Table S1).

We followed the protocol by Carleton et al. [49] to generate non-radioactive, digoxigenin (DIG)-labeled riboprobes. In brief, isolated plasmid DNA was restriction digested using BSSHII (New England Biolabs; Ipswich, MA), purified using Invitrogen’s PureLink PCR kit (Invitrogen; Carlsbad, CA), and run on an agarose gel to verify digestion, DNA content, and correct template size. Antisense probes were synthesized by incubating template with T3 RNA polymerase (Promega; Madison, WI) and DIG labeling mix (Roche Applied Science) for 2 h at 37 °C, purified using Sephadex G-50 columns and stored at − 80 °C until use.

### In situ hybridization and gene expression analysis

Non-radioactive in situ hybridizations to zebra finch sagittal brain sections were performed as in [49], using VectaMount Permanent Mounting solution for coverslipping. Expression for each gene was evaluated in sections from 2 to 3 different birds from a total of 10 birds. Brightfield microscopy was used to evaluate slides, and high-resolution images were acquired and uploaded to the Zebra Finch Brain Expression Atlas, ZEBrA [50]. Genes that showed greater or lesser mRNA signal within a song nucleus relative to their surround were designated as higher or lower expression, respectively. Incubation with no probes or antisense riboprobes for genes of known expression pattern (e.g. GAD2) were routinely included in the hybridizations as negative and positive controls, respectively. The hybridization conditions used have been previously shown not to generate significant signal to various sense-strand riboprobes.

### Microarray scoring

We also evaluated differential brain gene expression of ion channels based on data from four previous microarray experiments where the song nucleus of interest and a nearby region were microdissected using laser capture microscopy. The HVC dataset was generated using spotted glass ESTIMA:Song collection “20 k” cDNA microarrays as detailed in [48] and HVC was contrasted with the immediately ventral nidopallial shelf. Details on sample preparation, mRNA isolation, probe synthesis, and hybridization can be found in [51]. The RA, Area X, and nXIIts datasets were generated using Agilent microarrays spotted with oligonucleotides. RA and nXIIts samples were hybridized to the Duke University *Taeniopygia guttata* 45 K oligo array and Area X samples were hybridized to the 20 K Agilent-019785 Custom Zebra Finch Microarray. RA was contrasted to ventrolateral arcopallium, Area X was contrasted to ventral striato-pallidum, and nXIIts was contrasted with the supra-spinal medullary nucleus (SSP). Detailed methods of sample preparation, mRNA isolation, array hybridization, and first-pass analyses are available for RA [22], Area X [52], and nXIIts [53].

**Fig. 4:**
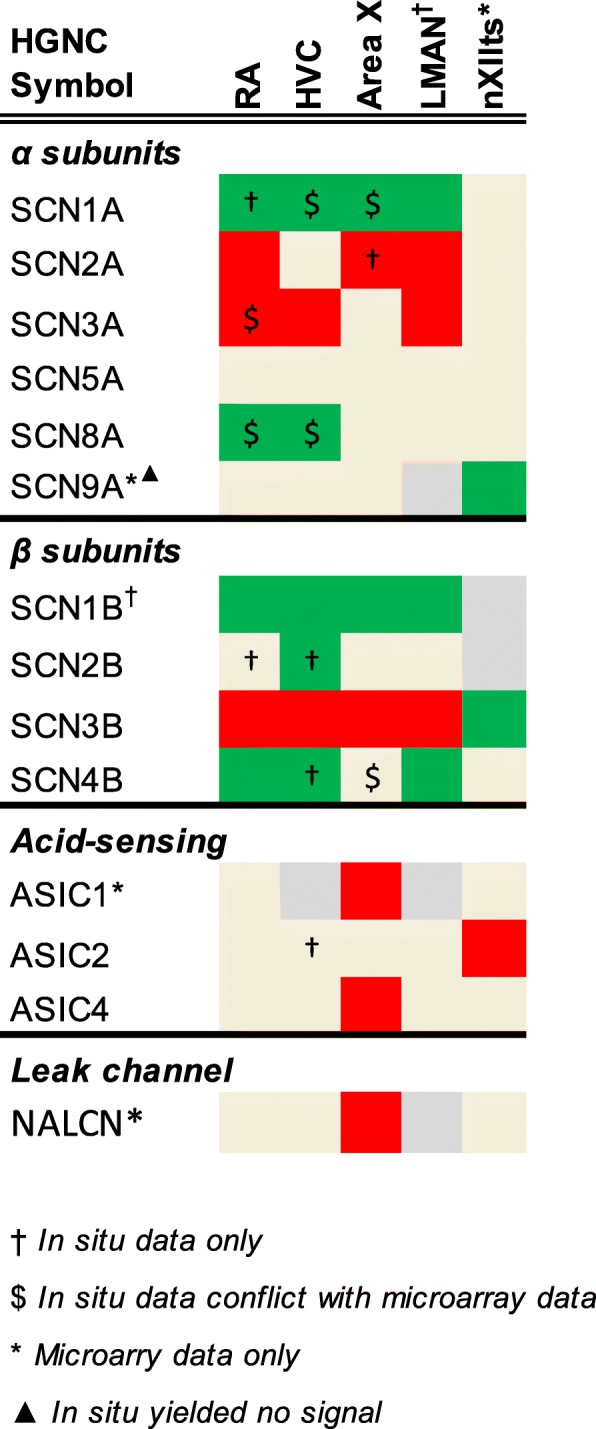
Regulation of sodium channel genes. Color shading indicates a gene that is higher (green), lower (red), or non-differential (shaded tan) in each song nucleus examined compared to the surrounding areas. Genes not assessed in a given nucleus are in grey. Gene expression was assessed by microarray and/or in situ hybridization

To determine the expression of ion channel and related genes in the zebra finch song system, these microarray datasets were reanalyzed as detailed in [54, 55]. Briefly, this involved mapping the oligonucleotide sequences onto taeGut1, visualizing them as a BED track uploaded to the UCSC Genome Browser, manually annotating oligo and cDNA entries, and using in situ hybridization data from the ZEBrA database for establishing validated significance criteria of differential expression. Here we evaluated the differential microarray data for each ion channel gene present in taeGut1 by navigating to the locus to confirm oligo and cDNA annotation and verify the microarray data outcome for that gene as higher, lower, or non-differential expression in each song nucleus analyzed compared to its contrasting region. This effort led to annotation of previously unannotated oligos or correction of mis-annotated ones, contributing to the manual curation efforts outlined in [54, 55]. Differential expression tables constructed in Excel are presented in Figs. 4 5, 6. When the microarray outcome was in disagreement with the in situ hybridization data, the latter designation was used as the final outcome (indicated by “$” in Figs. 4 5, 6). Some of these discrepancies were likely due to the song nuclei and comparison regions being in the same sagittal plane for in situs but not for microarrays (in the latter, RA was compared to ventrolateral arcopallium, and Area X was compared to ventromedial striato-pallidum). In some other cases, probes were not available for in situs, so expression data reflects microarray data only (indicated by asterisks in Figs. 4 5, 6).

**Fig. 5:**
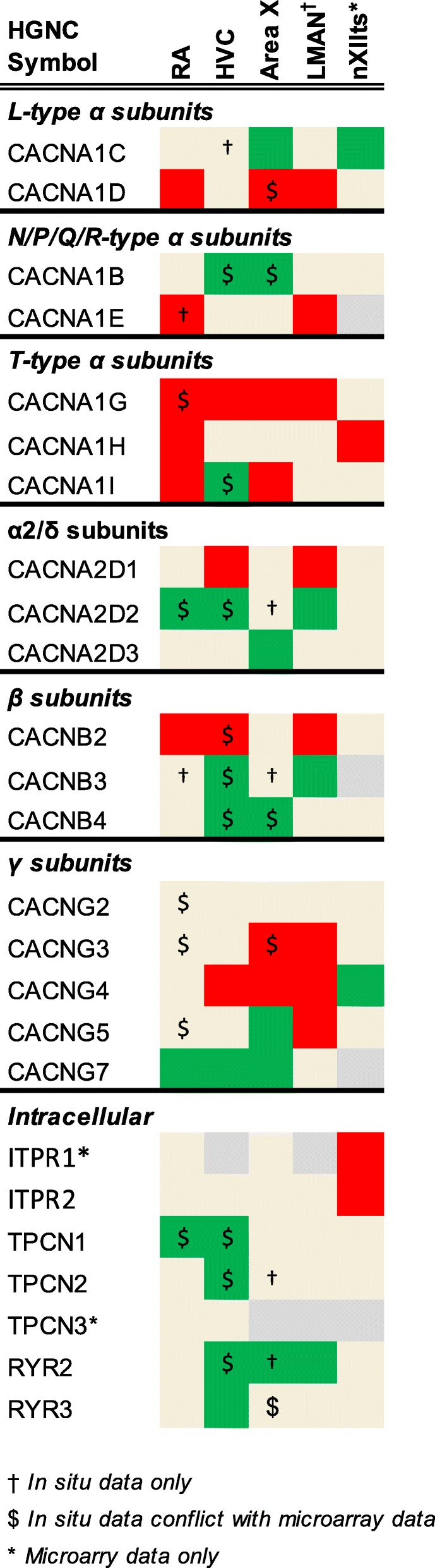
Regulation of calcium channel genes. Color shading indicates a gene that is higher (green), lower (red), or non-differential (shaded tan) in each song nucleus examined compared to the surrounding areas. Genes not assessed in a given nucleus are in grey. Gene expression was assessed by microarray and/or in situ hybridization

**Fig. 6:**
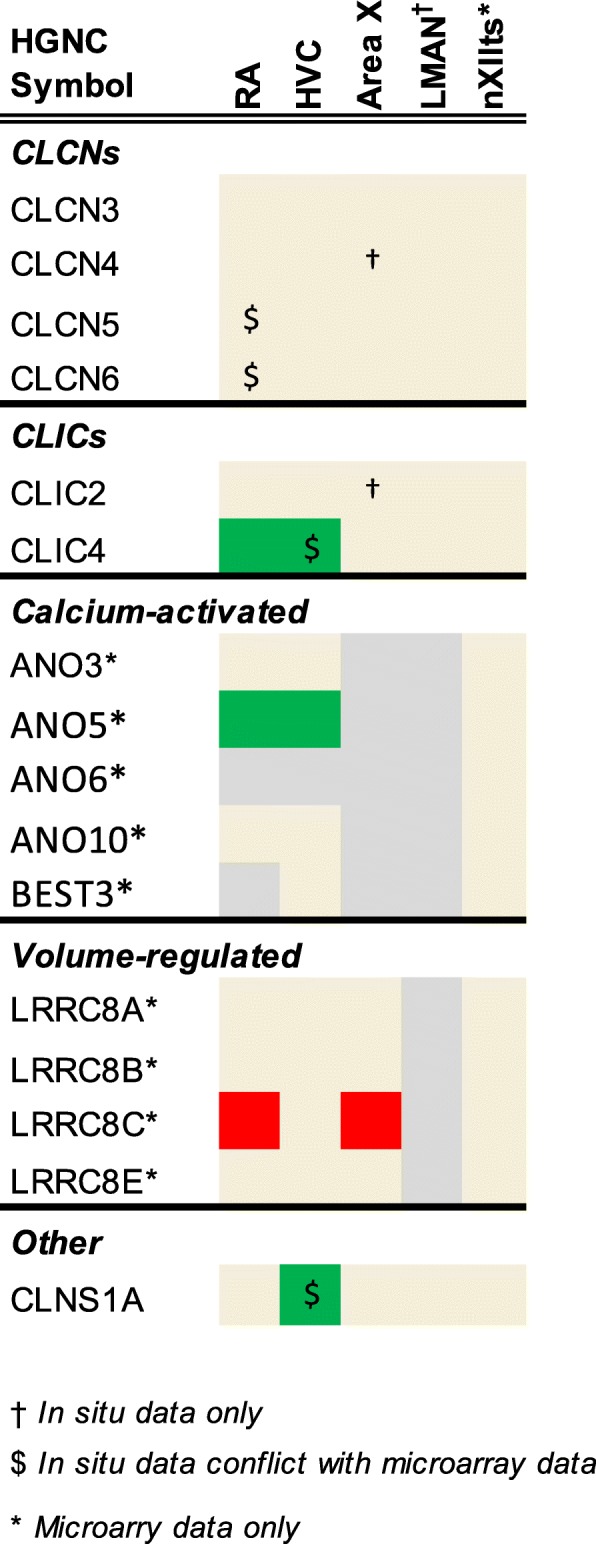
Regulation of chloride channel genes. Color shading indicates a gene that is higher (green), lower (red), or non-differential (shaded tan) in each song nucleus examined compared to the surrounding areas. Genes not assessed in a given nucleus are in grey. Gene expression was assessed by microarray and/or in situ hybridization

## Results

### Sodium, calcium, and chloride channel genes in the zebra finch genome

Genes that encode or modulate sodium, calcium, and chloride ion channels play important roles in determining the electrophysiological properties of brain circuits. In order to understand the potential contributions of these channels to functional circuits underlying vocal learning and production in songbirds, we started with a comprehensive analysis to define the full complement of these ion channel families in the zebra finch genome. Starting with a list of 108 ion channel genes previously identified in human plus one additional gene that lacks a human model (TPCN3), we used BLAT alignments and synteny analysis to identify orthologous loci in zebra finch and chicken. We identified 94 (23 sodium, 38 calcium, 33 chloride) of these genes in zebra finch (Table 1) and 98 in chicken (Additional file 1: Table S1), the latter encompassing four genes (BEST2, BEST4, CLCA1, and CLCA2) not found in zebra finch (Table 2 and Additional file 1: Table S2). Of the identified zebra finch genes, 54 were predicted and correctly annotated by Ensembl (taeGut3.2.4 release 95), 27 genes comprising 34 models were annotated as “novel genes” in Ensembl (indicated by a “^” in the “Zebra finch locus” column of Table 1), and 14 had no Ensembl predictions in taeGut1. Of these 14, four genes were found in taeGut1 through BLAT alignments of human or other species’ orthologs (indicated by a taeGut1 locus only in the “Zebra finch locus” column of Table 1.) The remaining 10 genes were not in taeGut1, but were identified in the recently released zebra finch PacBio assembly (indicated by a PacBio locus only in the “Zebra finch locus” column of Table 1) through alignments of orthologous models. For five of these genes only very partial alignments were found, suggesting gene truncation (indicated by a “▽” in Table 1.) In most cases we established orthology by demonstrating reciprocal top-scoring alignments and similar syntenies in birds and humans. For 8 genes, we linked the zebra finch synteny with human through phylogenetic relationships (indicated by a “$” in Table 1), but for 2 genes we could not fully link the synteny in zebra finch with that of human (indicated by a “#” in Table 1, detail provided in “Synteny notes” column of Additional file 1: Table S1). For a total of 14 genes in this study, we used orthologs from other species to find the locus in zebra finch (indicated by a “◆” in Table 1.) While no novel paralogs were found in zebra finch, we confirmed the presence of TPCN3 in birds and reptiles, a gene that is severely truncated in humans and chimp and completely missing from mouse and rat [56].

**Table 2:** Ion channel genes missing in the zebra finch genome

HGNC Symbol	Alternate names	Full name
*Missing in finches*		
BEST2	VMD2L1	bestrophin 2
*Missing in Passeriformes*		
CATSPER2	SPGF7, CATSPER	cation channel sperm associated 2
BEST4	VMD2L2	bestrophin 4
CLCA1	CaCC1	chloride channel accessory 1
CLCA2	CaCC3	chloride channel accessory 2
CLCA4	CaCC2	chloride channel accessory 4
*Missing in Neognathae*		
CATSPERG	C19orf15	cation channel sperm associated auxiliary subunit G
CATSPER1		cation channel sperm associated 1
CATSPER4		cation channel sperm associated 4
CLIC1	CL1C1	chloride intracellular channel 1
*Missing in all birds*		
CACNG6		calcium voltage-gated channel auxiliary subunit gamma 6
LRRC8E		leucine rich repeat containing 8 VRAC subunit E
*Unique to mammals*		
SCN7A	Nav2.1, Nav2.2, SCN6A	sodium channel, voltage-gated, type VII alpha subunit
CATSPERZ	TEX40, C11orf20	catsper channel auxiliary subunit zeta
CLCNKA or CLCNKB	CLCK1, ClC-K1; CLCKB, ClC-K2	chloride voltage-gated channel K A or B

For 15 mammalian genes we did not find orthologs in zebra finch, but we established a possible origin of the loss by exploring outgroup species (Table 2; details in Additional file 1: Table S2). These genes are discussed further in the context of their respective subfamilies. We note these conclusions about gene losses are tentative given that many avian genomes are incomplete and phylogenetic representation of sequenced genomes is somewhat limited.

### Sodium channel genes (Table 1A)

#### Sodium voltage-gated channel alpha (α) subunits (SCNxA):

The subunits encoded by these genes form the pore of voltage-gated sodium channels [34], which play important roles in the initiation and rising phase of action potential (AP) generation, and are critical for AP propagation [27]. This family originated from a singular ancestral form that has undergone multiple duplications and diversified broadly [57, 58]. We found 9 of the 10 mammalian SCNxA genes in zebra finch and chicken. The missing gene was SNC7A, which arose in mammals and presumably functions not as a voltage-gated channel but rather as a sodium sensor [59].

#### Sodium voltage-gated channel beta subunits (SCNxB):

Four genes encoding non-pore-forming, auxiliary β subunits have been described in mammals [60]. They are thought to modulate sodium currents by associating covalently (SCN1B and 3B) or non-covalently (SCN2B and 4B) with α subunits to form heteromeric complexes. We found all four β subunit genes in zebra finch and chicken, and note that in the latter, it was previously identified in a PacBio assembly [41] . SCN1B was missing from taeGut1 but limited evidence suggested its presence in songbirds [61]. We have now identified the complete gene and syntenic context in the PacBio assembly through ortholog alignments and verification of conserved synteny (Fig. 2 and Additional file 3). The synteny of SCN1B throughout avian phylogeny was difficult to resolve as many avian models were either on short scaffolds with little syntenic context or on scaffolds with many gaps, but we identified a consistent group of syntenic genes across lineages.

#### Acid-sensing ion channels (ASIC):

ASICs are proton-activated, nonselective cation channels that pass sodium, lithium, and potassium ions with the highest affinity for sodium [62]. All 5 mammalian ASIC genes were found in both zebra finch and chicken genomes.

#### Sodium leak channel (NALCN):

NALCN is a single gene that encodes a widely-expressed, nonselective, voltage-insensitive cation channel. This channel carries a persistent sodium leak current that sets excitability thresholds in virtually all neurons [63]. It is present in zebra finch and chicken genomes.

#### Epithelial sodium channels (SCNN):

Channel subunits in the SCNN family are encoded by four genes and drive active sodium reabsorption from extracellular fluid in epithelial tissues [64]. We found that all four genes are present in zebra finch and chicken genomes, confirming Hanukoglu et al. [65] and strengthening the evidence for orthology by confirming synteny.

### Calcium channel genes (Table 1B)

#### Calcium voltage-gated channel alpha (α) -1 subunits (CACNA1x):

Genes in this family encode the pore-forming α1 subunits that carry calcium across the plasma membrane [66]. In mammals, α1 subunits belong to three major subfamilies: Ca_v_1 (L-type) channels (4 genes), Ca_v_2 (P/Q-, N-, and R- type) channels (3 genes), and Ca_v_3 (T-type) channels (3 genes). All genes were found in zebra finch and chicken, with CACNA1A and CACNA1F in the PacBio assembly.

#### Calcium voltage-gated channel alpha-2-delta (α2/δ) subunits (CACNA2Dx):

Calcium channels are modified by non-conducting subunits, including α2 and δ. α2/δ genes are unique in that each gene codes for both the α2 and δ subunits: α2 is an extracellular glycoprotein that forms a disulfide linkage to the δ subunit, and the δ subunit keeps the complex tethered to the plasma membrane [67]. All four human α2/δ genes were found in zebra finch and chicken.

#### Calcium voltage-gated channel auxiliary beta (β) subunits (CACNBx):

These modulatory subunits are entirely intracellular and attached to the calcium channel complex through binding sites on the α1 subunit [68]. All four human genes were found in zebra finch and chicken. CACNB1 in zebra finch was found only in the PacBio assembly.

#### Calcium channel auxiliary gamma (γ) subunits (CACNGx):

Gamma subunits are glycoproteins with four transmembrane domains that associate with calcium channel complexes in the membrane [69]. Of the eight human genes, three (CACNG6, CACNG7, and CACNG8) occur in a syntenic block within a region of human Chr19 that is largely missing in birds, possibly due to chromosomal rearrangements in ancestral archosaurs and/or dinosaurs [61]. Subsequent assessments have identified CACNG7 and CACNG8 but not CACNG6 in the galGal5 chicken assembly [41]. Here we have confirmed with high confidence that CACNG6 is missing in zebra finch and other avian lineages based on BLAST searches and synteny verification of bird genomes including chicken and zebra finch PacBio assemblies (Fig. 3), suggesting an ancestral avian loss. We also found evidence that CACNG7 is present and likely complete in zebra finch, while CACNG8 is present but appears truncated in both zebra finch and chicken.

#### Sperm associated cation channels (CATPSER):

This family encodes four α subunits (CATSPER1–4) and five auxiliary subunits (CATSPER B, D, E, G, & Z) essential to sperm activation and motility [70, 71]. In zebra finch, we found fragments of CATSPER3, B, D, and E, but no trace of CATSPER1, 2, 4, G and Z. Chicken shares the same pattern, but retains fragments of CATSPER2. Because CATSPER1, − 4, and -G are present in ratites, their absence in zebra finch and chicken appears to reflect loss in Neognaths. Our conclusions are partially consistent with Cai et al. [72], Chung et al. [70], and Warren et al. [41], but due to the higher quality PacBio scaffolds and examination of additional birds, we provide stronger evidence with regards to truncation and phylogenetic patterns of gene loss. We note that the chicken gene annotated as CATSPER2 in NCBI (Gene ID: 395985) is misannotated – the correct annotation is CACNA1S.

#### Other calcium channel genes (ITPR, RYR, TPCN):

Other calcium channels may impact neuronal function by regulating internal calcium stores, including inositol 1,4,5-trisphosphate receptors (ITPR), ryanodine receptors (RYR), and two-pore channels (TPCN) [56, 73, 74]. Each family has 3 genes, all of which were identified in zebra finch and chicken. TPCN3 appears to have been lost in rodents and truncated in primates, including humans [56]. Interestingly, the truncated gene appears to have been duplicated in human, as can be seen in the UCSC Browser (GRCh38/hg38 chr2:109947776–109,974,086 and chr2:110397405–110,423,734).

### Chloride Channel genes (Table 1C)

#### Voltage-dependent chloride channels (CLCN):

This family is composed of channels that open and conduct chloride ions in response to changes in membrane potential [36]. Mammals have 10 CLCN genes, of which 9 were identified in zebra finch and chicken. Two adjacent human genes - CLCNKA and CLCNKB - have high sequence identity and appear to be the result of a duplication specific to mammals. Both chicken and finch have a single gene at this locus that mirrors the ancestral condition. Because of their high similarity, sequence alignments could not resolve which human paralog is the ancestral form present in birds or reptiles. As the nomenclature in ancestral species is inconsistent, we suggest the name “CLCNK” for the single gene found at this locus in birds and reptiles.

#### Chloride intracellular channels (CLIC):

These channels are integrated into the membranes of organelles and participate in processes like membrane trafficking and cytoskeleton dynamics [75]. They can also exist in a soluble state in the cytoplasm, the function of which is poorly understood. Of the 7 functional genes in mammals, we found all but CLIC1 in chicken and zebra finch. We could not conclusively establish a CLIC1 loss because the syntenic genes are at the ends of different scaffolds in both zebra finch and chicken PacBio assemblies. It thus remains possible that CLIC1 is not in these assemblies due to incompleteness of the scaffolds. Consistent with this possibility, CLIC1 and syntenic genes are present in alligator and in kiwi (a ratite). Alternatively, the gene was lost in neognaths, as kiwi is currently the only bird with a detectable CLIC1.

#### Calcium-activated chloride channels (ANO, BEST):

Some anoctamin (ANO1 & ANO2) and bestrohpin (BEST2) genes encode channels that conduct chloride currents in response to surges in intracellular calcium, but the function of most other family members remains speculative [76, 77]. Mammals have 10 ANO genes, of which we found 9 in chicken and zebra finch, and 4 BEST genes, of which we found 4 in chicken and 2 in zebra finch.

ANO7 lacks models in zebra finch and chicken, but traces were detected by aligning orthologs to the zebra finch and chicken PacBio assemblies. The synteny in these and other birds (kiwi, goose, golden eagle, and rifleman) is conserved with mammals and alligators. Given that only a few 3′ exons from orthologous models of this gene aligned to the chicken and zebra finch PacBio assemblies, combined with the lack of EST-based expression evidence, we conclude that ANO7 has likely become a pseudogene in some avian lineages, including oscines and galliformes.

BEST4 is present and complete in galliforms (chicken) and psittaciformes (budgie), but appears pseudogenized in passerines, as only short segments of the 5′ and 3′ UTR regions were detected in zebra finch (PacBio), Bengalese finch, great tit, and starling. While BEST2 seems complete in chicken and songbirds like Tibetan tit and starling, we did not detect it at the corresponding locus in the zebra finch PacBio assembly. The upstream genes are in zebra finch similarly as in other birds, reptiles, and mammals, while the genes downstream of this locus reflect a synteny shared by Tibetan tit and starling that is presumably unique to songbirds. Because there are no BEST2 models and syntenic genes are isolated on short scaffolds in Bengalese and medium ground finch, we cannot distinguish between a loss in the finch lineage and a zebra finch-specific loss. Similar to CACNG6/7/8, BEST2 is located in a region of human chromosome 19 associated with extensive rearrangements and gene losses in birds [61].

#### Chloride channel accessory subunits (CLCA):

Mammals have several non-pore-forming accessory subunits with a broad functional repertoire that includes modulating chloride channels, cell adhesion, and tumor suppression [78]. In humans, three functional CLCAs are in a syntenic cluster (CLCA1, CLCA2, and CLCA4) along with a pseudogene (CLCA3P). We found at least one CLCA gene with conserved synteny in psittaciformes (budgie), accipitriformes (eagles), and more basal birds (chicken, guinea fowl, and tinamou). In zebra finch and other passerines (e.g. golden-collared manakin), the immediately syntenic genes were adjacent to one another, with no trace of CLCA and no gaps in the intergenic region. From this, we conclude that CLCA genes were likely lost in Passeriformes.

#### Volume-regulated chloride channels (LRRC8):

LRRC8 channels are activated by cell swelling and may influence extracellular signaling by transporting neurotransmitters [79, 80]. Mammals have five LRRC8 subunits, and all but one (LRRC8E) were found in zebra finch and chicken. This gene appears to have been lost in birds [81].

#### Other chloride channel genes:

Cystic fibrosis transmembrane conductance regulator (CFTR) is a chloride channel gated by ATP [82]. Chloride nucleotide-sensitive channel 1A (CLNS1A) is expressed at relatively high levels in human [38] and mouse [40] brain tissue, and strongly implicated in cell volume regulation [83]. Both genes are present in zebra finch and chicken.

### Evaluating zebra finch ion channel gene models

While examining zebra finch gene orthology, we noticed that many human models were noticeably longer than the corresponding zebra finch Ensembl models, suggesting that the latter may be incomplete predictions. To record this difference, we calculated a ratio (Additional file 1: Table S1 and 3A) of zebra finch to human transcript model length, and found that 28 of the 80 existing zebra finch Ensembl models were less than 90% of the human length, and 19 were less than 75%, even after summing the lengths of partial models, when present. To further evaluate model completeness, we compared the alignments of orthologous chicken and zebra finch Ensembl models at each gene locus in taeGut1 (*n* = 79 genes with an Ensembl model in both species). Because Ensembl chicken models are from a higher quality genome and are generally more complete than zebra finch models, this method revealed sequence blocks where the chicken model aligned but that are not present in the zebra finch Ensembl models (example in Additional file 5). We recovered these additional sequences for 66 of the 79 genes analyzed (Additional file 1: Table S1, “Additional bases recovered” > 0), and created a taeGut1 BED track for their visualization in the UCSC Browser (Additional file 6). We also calculated a percent of post-recovery length (Additional file 1: Table S1; Additional file 4B) and found 36 genes for which the original model length was less than 90% of the post-recovery length (indicated by a “*” in the “Zebra finch locus” column of Table 1), and 16 that were less than 75%. This effort demonstrates that many zebra finch Ensembl models are incomplete, but additional genomic sequence related to those genes exists and should be taken into account in future studies.

### Differential expression of sodium, calcium, and chloride channel genes in the song system

**Fig. 7:**
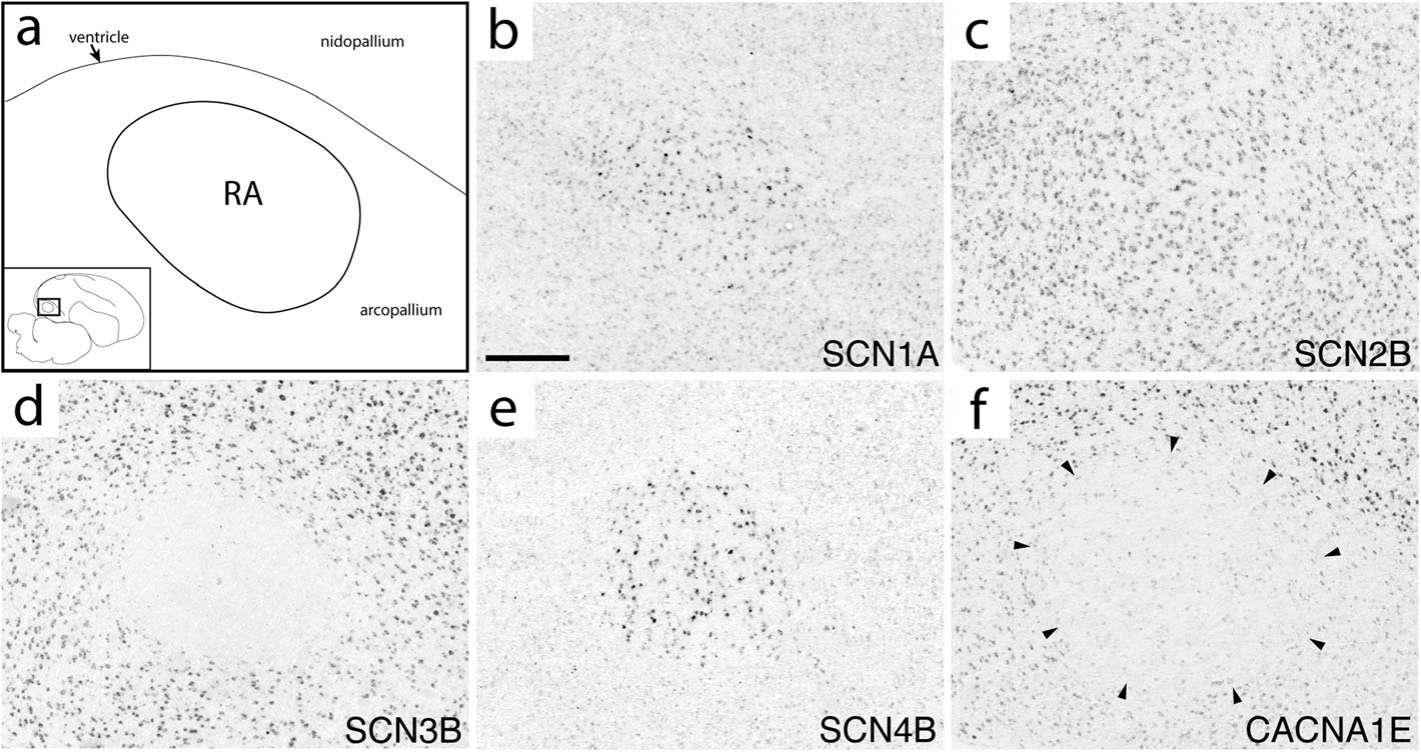
Expression of select ion channel genes in RA. (**a**) Camera lucida drawing depicting RA and surrounding arcopallium & nidopallium in the parasagittal plane (approximate location is indicated in inset; the dorsal arcopallial lamina is depicted by thin line.) (**b**-**f**) Representative in situ hybridization photomicrographs of select ion channel genes that show differential (**b**, **d**-**f**) and non-differential (**c**) expression in RA compared to the surrounding arcopallium. Gene abbreviations are given in Table 1. Scale bar: 500 μm

To assess the expression of ion channel genes in the song system of adult male zebra finches, we analyzed previously generated microarray data and conducted in situ hybridizations on brain sections containing the 4 major telencephalic song nuclei (HVC, RA, LMAN, and Area X), and a brainstem motor nucleus, nXIIts. Because our focus was on vocal control circuitry, not all brain areas of relevance for vocal learning (such as auditory forebrain) were assessed. This analysis encompassed 55 genes (14 Na, 25 Ca, 16 Cl) that were part of the microarrays and/or for which cDNA clones were available in zebra finch. Summaries of the data are presented in Figs. 4, 5, 6, and robust examples of differential in situ patterns are presented in Figs. 7 8, 9, 10, and 11 Additional file 7. In situ images can also be accessed through ZEBrA [50]. For a few genes, in situs also revealed differential expression in DLM and Uva. For all genes analyzed, the terms higher, lower and non-differential below refer to expression compared to the respective surrounds (nidopallium for HVC, LMAN and NIf, arcopallium for RA, striatum for Area X, dorsal thamalus for DLM and Uva, rostral medulla for nXIIts).

**Fig. 8:**
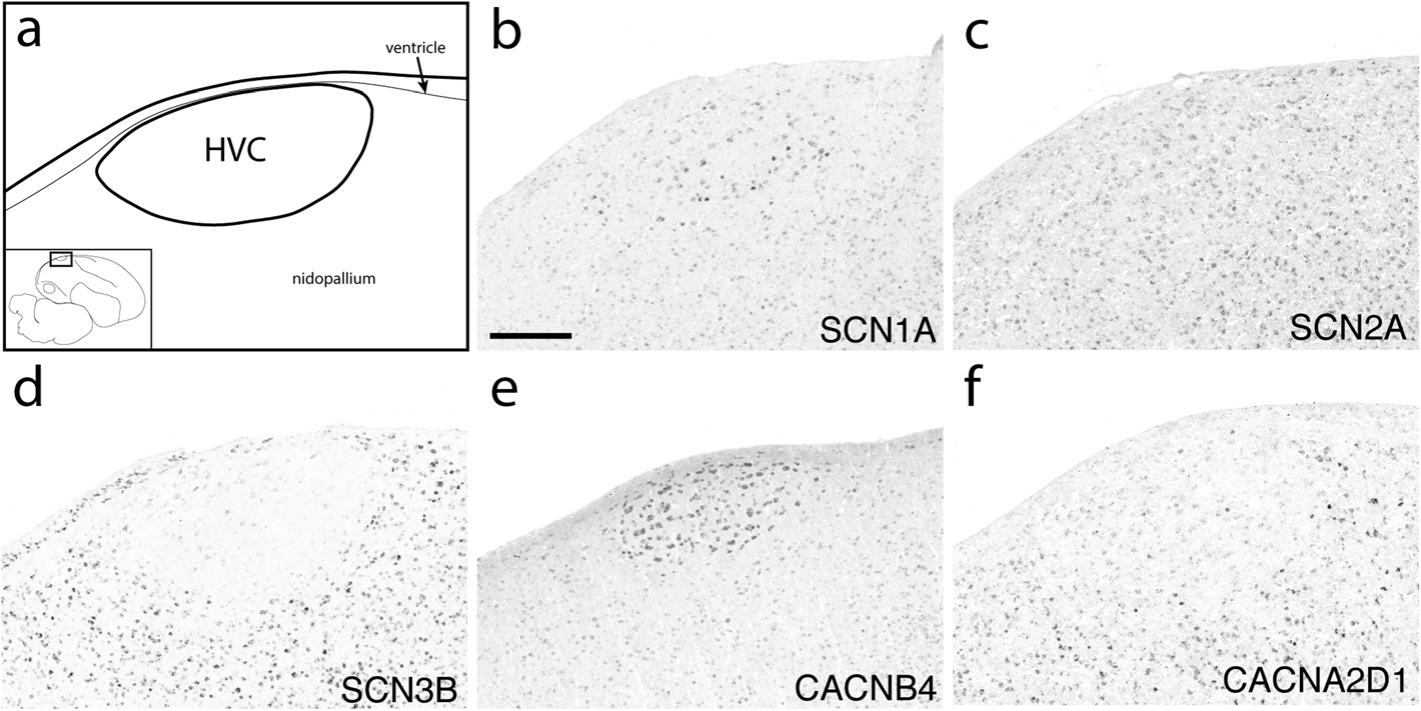
Expression of select ion channel genes in HVC. (**a**) Camera lucida drawing depicting HVC and surrounding nidopallium in the parasagittal plane (approximate location is indicated in inset.) (**b**-**f**) Representative in situ hybridization photomicrographs of select ion channel genes that show differential (**b**, **d**-**f**) and non-differential (**c**) expression in HVC compared to the adjacent nidopallium. Gene abbreviations are given in Table 1. Scale bar: 500 μm

**Fig. 9:**
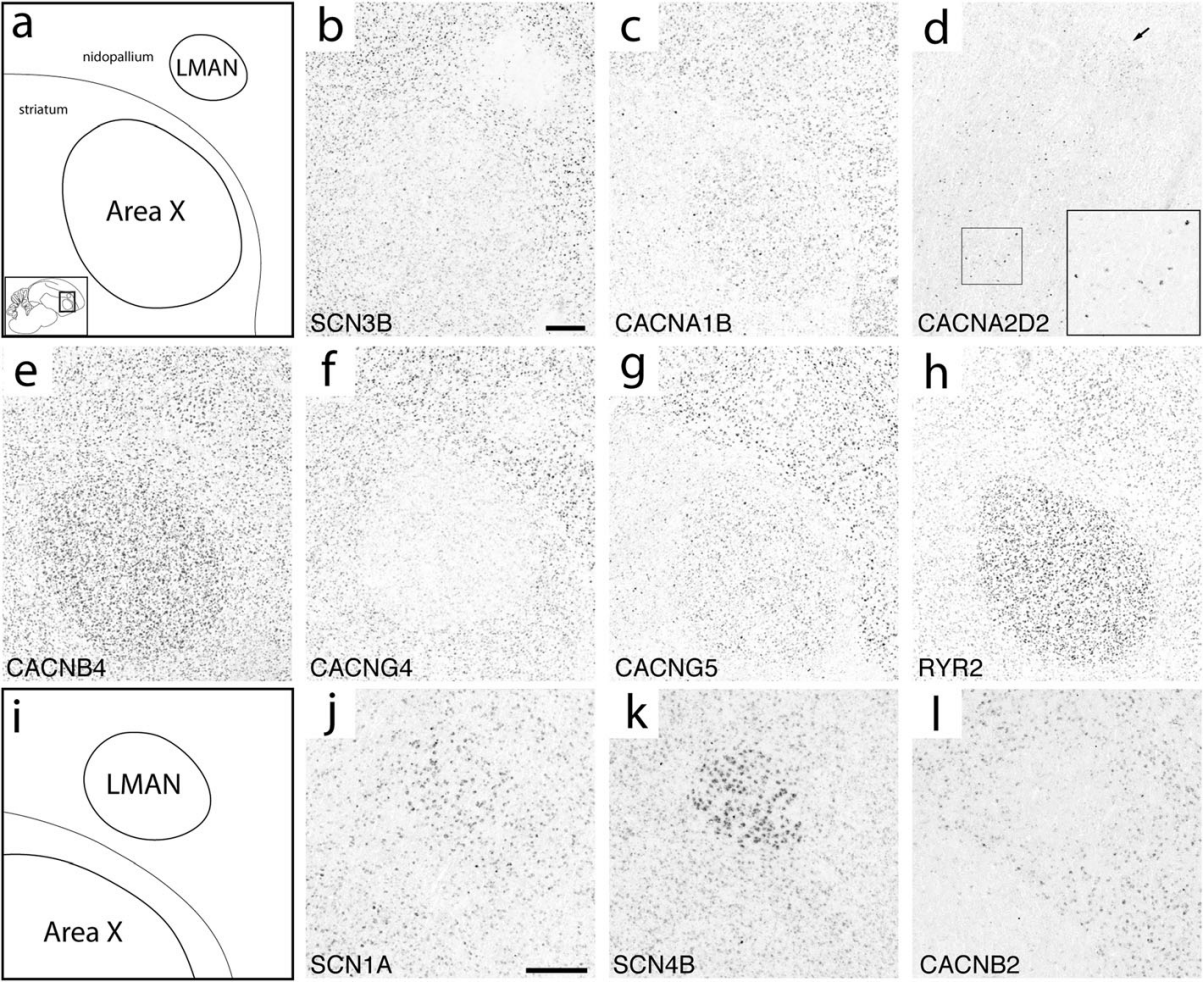
Expression of select ion channel genes in LMAN and Area X. (**a**) Camera lucida drawing depicting anterior portions of the nidopallium and medial striatum including song nuclei LMAN and Area X in the parasagittal plane (approximate location is indicated in inset; the pallial-subpallial lamina is depicted by thin line). (**b**-**h**) Representative in situ hybridization photomicrographs of select ion channel genes that show differential expression in Area X (all panels except **d**) and LMAN (**b**, **d**, **f**-**h**) compared to adjacent regions. Arrow in (**d**) indicates LMAN and inset shows enhanced labeling in a population of sparse cells from the indicated region of Area X. (**i**) Camera lucida drawing depicting LMAN and dorsal Area X at same level as in (**a**). (**j**-**l**) Representative in situ hybridization photomicrographs of select ion channel genes that show differential regulation in LMAN compared to the adjacent nidopallium. Note the sparse, darkly stained cells in Area X in panel (**j**). Gene abbreviations are given in Table 1. Scale bars for each panel series are 500 μm

**Fig. 10:**
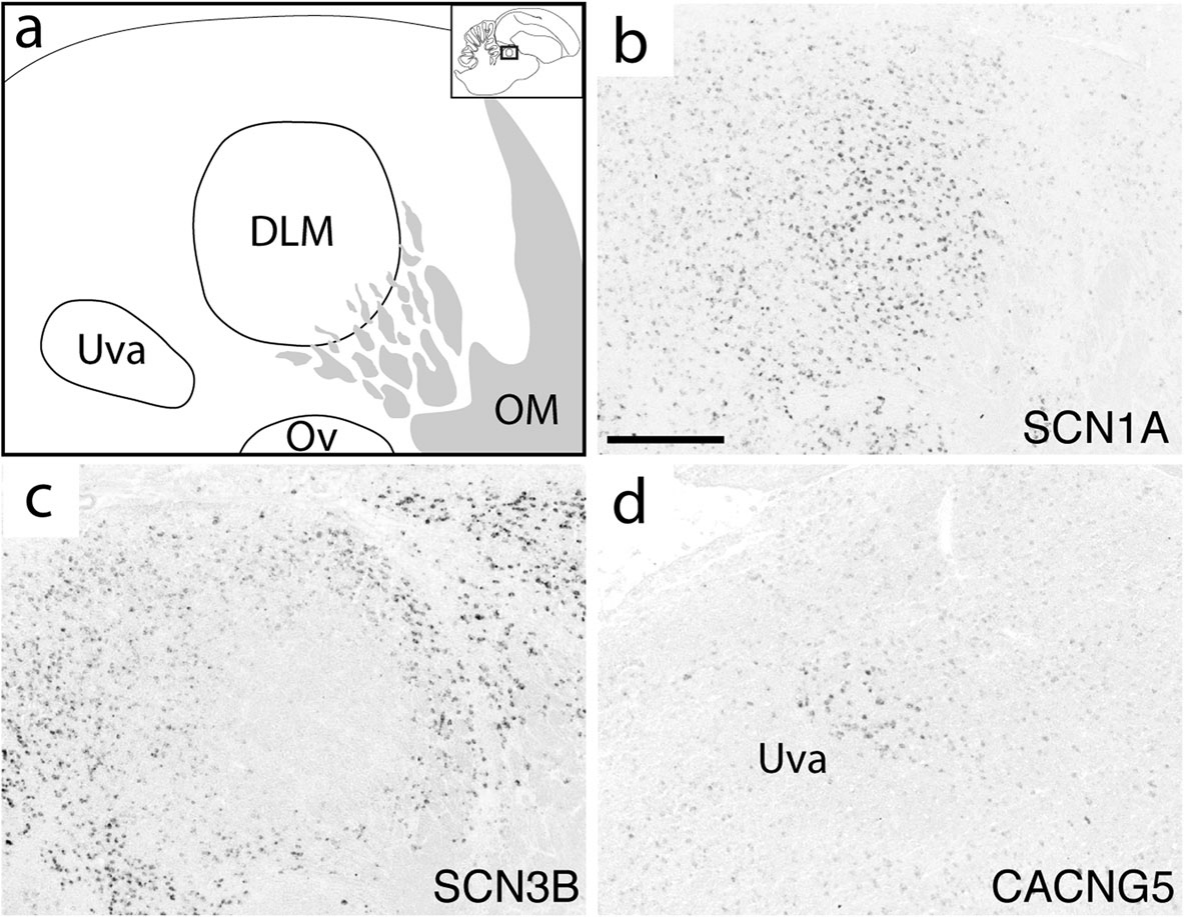
Expression of select ion channel genes in DLM and Uva. (**a**) Camera lucida drawing depicting DLM, Uva, and surrounding thalamic areas in the sagittal plane (approximate location is indicated in inset.) (**b**-**d**) Representative in situ hybridization photomicrographs of select ion channel genes that show differential regulation in DLM (**b**, **c**) or Uva (**d**). Gene abbreviations are given in Table 1. Scale bar: 500 μm

**Fig. 11:**
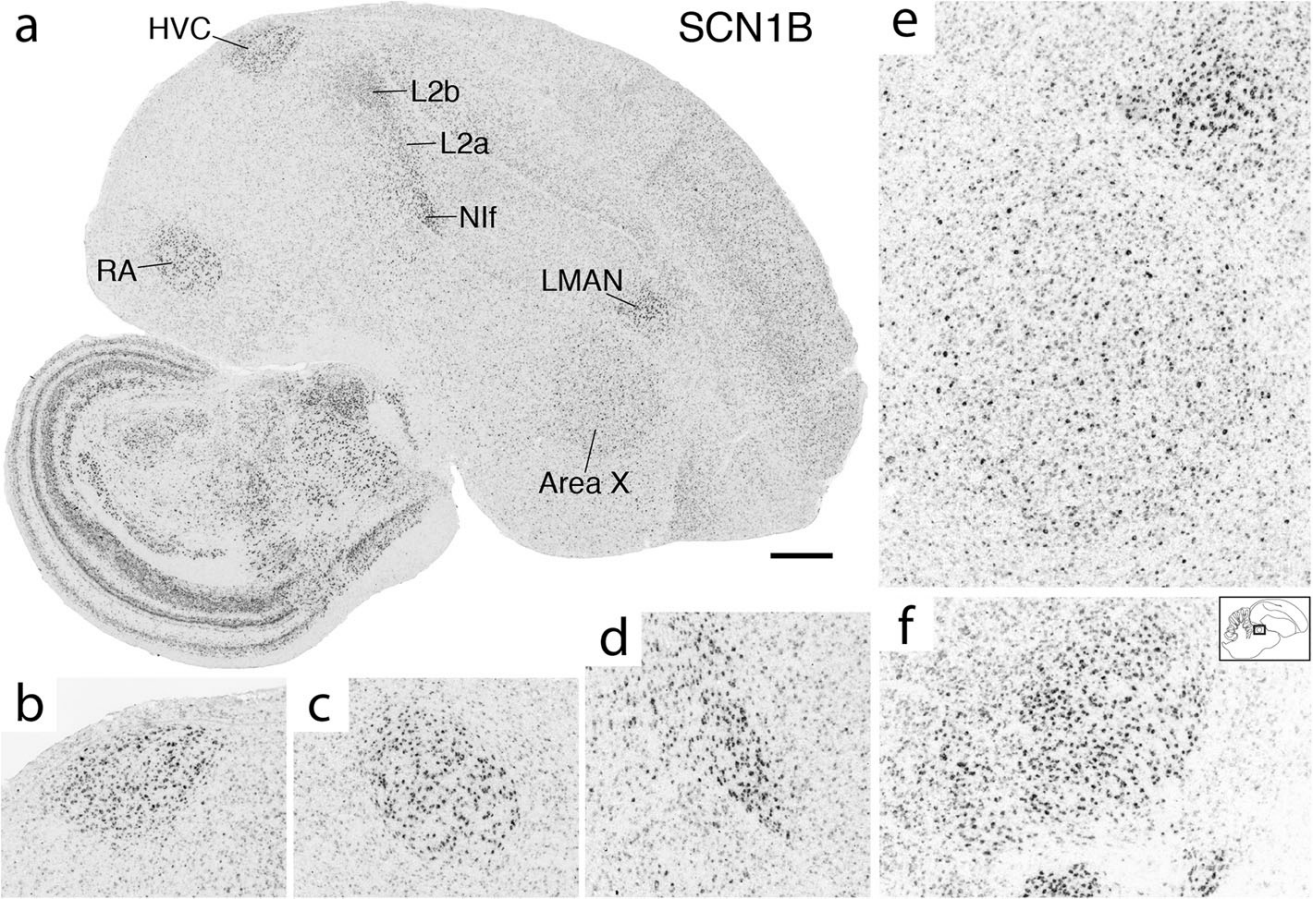
SCN1B is strongly upregulated in multiple song system nuclei. (**a**) In situ hybridization photomicrograph of a whole parasagittal section. Individual song nuclei in (**a**) are shown in detail in (**b**) HVC, (**c**) RA, (**d**) NIf, and (**e**) Area X and LMAN. SCN1B is also noticeably upregulated in Field L2a and L2b as shown in (**a**). (**f**) SCN1B upregulation in DLM (approximate location shown in inset and comparable to Fig. 10). Scale bar in (**a**): 1 mm

#### Sodium channels

The four alpha subunits with the highest brain expression in mammals (SCN1A/2A/3A/8A) showed varied patterns within the song system (Fig. 4). SCN1A was higher in RA (Fig. 7b), HVC (Fig. 8b), LMAN (Fig. 9j), Area X, and DLM (Fig. 10b). SCN2A was lower in RA (Additional file 7), Area X, LMAN, and DLM (Fig. 10c), but non-differential in HVC (Fig. 8c). SCN3A was lower in RA (Fig. 7d), HVC (Additional file 7), and LMAN. SCN8A was higher in RA (Additional file 7) and HVC (Additional file 7). In contrast, SCN5A was non-differential in all nuclei, and SCN9A was non-differential except in nXIIts where it was higher and the only alpha subunit to show differential expression in this nucleus.

SCN1B showed higher expression in all telencephalic nuclei, including NIf, as well as in DLM (Fig. 11). In contrast, SCN2B was non-differential across song nuclei (e.g. RA in Fig. 7c) except for higher expression in HVC (Fig. 8c). SCN3B was an exquisite negative marker of all telencephalic song nuclei (Figs. 7d, 8d, 9b, and 10c), but higher in nXIIts. Interestingly, SCN3B was highly expressed in a sparse cell population in Area X (Additional file 7). SCN4B was higher in RA (Fig. 7e), HVC, and LMAN (Fig. 9k). Area X showed lower expression of ASIC1 and ASIC4, and ASIC2 was lower in nXIIts. NALCN, the only sodium channel that is a leak channel, showed lower expression in Area X.

#### Calcium channels

Among Ca_v_1s, CACNA1C was higher in Area X and nXIIts, while CACNA1D was lower in RA, Area X, and LMAN. Among Ca_v_2s, CACNA1E was lower in RA (Fig. 7f) and LMAN, while CACNA1B was higher in HVC and Area X (Fig. 9c) where it was particularly strong in a sparse population of Area X cells (Additional file 7). Differential expression of Ca_v_3’s was biased toward lower expression, except for higher expression of CACNA1I (Ca_v_3.3) in HVC (Additional file 7), the only calcium channel α subunit to exhibit opposing expression in two different song nuclei. All three Ca_v_3 channels were lower in RA, the only nucleus to show differential expression in the same direction of every gene within a channel subfamily.

CACNA2D1 and CACNA2D2 were complementary in their differential expression: CACNA2D1 was lower in HVC (Fig. 8f) and LMAN, and CACNA2D2 higher in HVC and LMAN (Fig. 9d). In Area X, CACNA2D2 was non-differential but strongly expressed in a sparse cell population, with a similar pattern throughout the striatum (Fig. 9d). CACNA2D2 was also higher in RA, and CACNA2D3 higher only in Area X.

The only calcium channel β subunit to show lower expression was CACNB2, which was turned down in RA, HVC (Additional file 7), and LMAN (Fig. 9l), but higher in DLM (Additional file 7). CACNB2 was also generally lower throughout much of the anterior striatum, but a sparse cell population showed enhanced expression (Additional file 7). CACNB3 was higher in HVC and LMAN and CACNB4 higher in HVC (Fig. 8e) and Area X (Fig. 9e).

Differential expression of γ subunits in the song system was predominantly in the AFP. CACNG5 was lower in LMAN (Fig. 9g) and higher in Area X, and CACNG3 was lower in Area X and LMAN. CACNG5 also showed enhanced expression in a sparse cell population of Area X (Additional file 7), and in Uva (Fig. 10d). CACNG4 was lower in HVC, LMAN and Area X (Fig. 9f), but higher in nXIIts. All γ subunit genes examined were non-differential in RA (e.g. Additional file 7), except for CACNG7, which was higher in RA as well as in HVC and Area X.

Differential expression for TPCN and RYR genes was always higher, including RYR2 in HVC (Additional file 7), Area X and LMAN (Fig. 9h), and RYR3, TPCN − 1 and − 2 in HVC. Only nXIIts showed differential expression of ITPRs, which were lower.

#### Chloride channels

The majority of identified chloride channels lacked zebra finch ESTs. Among assessed genes, most were non-differential, except for three genes with higher expression - ANO5, CLIC4, and CLNS1A - which were all higher in HVC, with ANO5 and CLIC4 also higher in RA, and one gene with lower expression, LRRC8, in RA and Area X. Thus, differential expression of chloride channel genes was biased toward nuclei of the DMP and higher expression.

## Discussion

In spite of the wealth of information on song system connectivity and neurophysiology, an understanding of the molecular basis for the intrinsic electrophysiological properties of its constituent neurons is lacking. Here we report on the genomics and song system expression of sodium, calcium, and chloride ion channel genes in the zebra finch, including pore-forming and modulatory subunits. This effort annotated 34 “novel gene” Ensembl models, detected additional sequences for several partial gene models in taeGut1, and established what we believe is the full complement of these gene families in zebra finch and chicken, with no novel paralogs detected. PacBio assemblies were particularly helpful to: (1) identify previously undetected genes; (2) confirm with high confidence gene truncation; (3) define orthology based on synteny; (4) suggest a likely phylogenetic path to previously reported avian gene losses or truncations [41, 61]. A large number of zebra finch gene models are partial relative to human and chicken models, but we detected additional sequences in taeGut1 that could expand those models. Incomplete gene predictions (indicated by “*” in Table 1) cast doubt on “off-the-shelf” dN/dS values from automated algorithms. Thus, conclusions regarding positive selection or rapid gene evolution in these families (e.g. [42, 84, 85]) should be treated as provisional until more complete gene models are available.

Each song nucleus examined differentially expresses a unique complement of ion channels, suggesting that diverse expression of these genes is fundamental to shaping excitability within the song system. With few exceptions, differential expression of individual genes was in the same direction across nuclei. Area X and HVC showed enhanced expression of more calcium channel genes than other song nuclei, suggesting a more prominent role of calcium currents in governing electrophysiological properties of the AFP. In contrast, chloride channel genes were mostly non-differential. Compared to other nuclei, nXIIts differentially expressed the fewest number of genes. We note that most genes that lacked brain expression evidence in zebra finch (e.g. SCN4A, CACNG1, and CLCN1) are primarily expressed in non-neural tissues (e.g. skeletal muscle) in other organisms [38, 39].

Some genes were strongly expressed in sparse cells and might contribute to defining the unique neuronal populations reported in Area X [12, 86, 87], HVC [8, 13], and RA [9]. We acknowledge, however, that several of the genes in this study participate in processes other than excitability (e.g. cell signaling and metabolism), and expression in specific cell types or non-neuronal cells (e.g. glia) was not assessed. Furthermore, protein-based assays and coexpression analysis are needed to determine the subcellular localization of the channels and their possible interactions. We did not evaluate splice variants, but note that in mammals, genes like T-type calcium channels give rise to variants with distinct gating properties and spatiotemporal regulation [88]. We also note that while differential expression provides clues as to regulatory mechanisms, non-differentially expressed ion channels may still contribute to excitability. Below we discuss the main implications of the brain expression findings with regards to gene families.

### Sodium channels

Based on its known role in in mammals, the higher expression of SCN1A (Nav1.1) in all major song nuclei suggests an essential function of somatodendritic signal integration and action potential (AP) thresholding in the song system [89]. SCN1A also confers fast spiking in a diversity of GABAergic interneurons [90–92]. Because in most nuclei SCN1A was enhanced in small, sparse cells reminiscent of interneurons, the enhanced expression of SCN1A may contribute to the fast-spiking behavior of interneurons that has been described in multiple song nuclei [8–11, 86]. In mammals, SCN8A (Nav1.6) facilitates AP initiation and propagation [89, 93–95]. Its higher expression in RA and HVC could thus reinforce rapid and reliable axonal propagation in the DMP. SCN3A (Nav1.3) brain expression is high early in mammalian development but is progressively replaced by SCN1A, SCN2A, and SCN8A [96–98]. In adult zebra finch, SCN3A is widely expressed throughout the brain but lower in RA, HVC and LMAN - a species discrepancy that highlights the diversity in sodium channel expression across phylogenies.

β subunits alter cell surface expression, gating properties, and voltage sensitivities of α subunits [60], and high levels of β subunits are likely necessary to shuttle and modulate a high volume of voltage-gated sodium channels. Thus, it is not surprising that some α and β subunits were highly expressed within the same song nuclei. In mammals, SCN1B increases the surface expression and current densities of α subunits [60, 99, 100]. It also supports resurgent sodium currents that enable high-frequency firing [101], and reinforces AP initiation by targeting SCN8A to the axon initial segment [93]. Additionally, SCN1B can modulate voltage-gated potassium (Kv) family channels [102–104], several of which are higher in multiple song nuclei, including Kv1.1, Kv4.2, and Kv4.3 [1]. Collectively these features suggest that SCN1B may be an important regulator of neuronal excitability in song nuclei by supporting fast, reliable spiking. HVC was the only nucleus to show higher expression of both SCN1B and SCN2B, consistent with the higher expression of α subunits in HVC, and the observation that the sodium current-boosting effects of SCN2B require SCN1B coexpression [60, 99].

SCN4B induces resurgent sodium current, an important adaptation for neurons that exhibit high-frequency firing [95, 105–109]. Its high expression suggests that resurgent currents might support the high-frequency and bursty firing characteristic of neurons in RA [110, 111], HVC [112, 113], and LMAN [114]. SCN4B also supports persistent sodium currents [106], which might account for the non-inactivating sodium conductances of HVC neurons [4]. While the effects of SCN3B on excitability are inconsistent [115–120], its high brain expression in adult zebra finches contrasts with rodents, where expression is high during development and lower in adulthood [121]. Much like SCN3A, this stark divergence exemplifies how gene expression patterns can differ in birds versus mammals. As for ASICs, little is known about the role of acid signaling in neurotransmission, although there is evidence of their role in dendritic spine remodeling [62]. Lastly, leak currents carried by NALCN play roles in rhythmic and spontaneous firing [63, 122], thus NALCN differential expression might contribute to the heterogeneity in spontaneous firing rates across Area X cell types [11].

### Calcium channels

Calcium channels influence input processing in dendrites and signal transmission in synaptic terminals, which links them to plasticity mechanisms that serve learning and memory [66]. Voltage-gated calcium channels (Cav’s) are vital to dendritic processing, burst firing, and neurotransmitter release. Among pore-forming alpha subunits, L-type (Cav1) channels conduct large, long-lasting currents in response to strong depolarization [123]. The higher expression of the L-type channel CACNA1C suggests that integration of synaptic input is important in Area X and nXIIts. CACNA1C can also associate with BK channel KCa1.1 (KCNMA1) to modulate repolarization and spiking frequency [124, 125]. While not differential, KCNMA1 is expressed in Area X [33] and may interact with CACNA1C to shape repolarization and repetitive firing. Compared to CACNA1C, CACNA1D activates at lower membrane potentials and is slower to inactivate [126]. Thus, its lower expression may refine the timing and restrict the duration of depolarization-induced calcium influx in RA, Area X, and LMAN.

T-type (Cav3) channels create low-threshold calcium potentials that are integral to driving rebound and burst firing in neurons [88, 123, 127]. The lower expression of all T-type channels in RA is intriguing given that low expression of T-type channels is associated with single or tonic AP firing [88], yet RA neurons exhibit bursty, high-frequency firing [110, 111, 128]. This suggests that high-frequency firing in RA might instead be driven by voltage-gated sodium channel beta subunits (SCN1B, SCN4B) and/or potassium channel subunits like Kv3.1 [27, 129], which show high expression in RA [33, 50], or by hyperpolarization-activated (HCN; I_h_) channels as suggested by electrophysiology studies of HVC [4]. HVC was the only nucleus to highly express a T-type channel (CACNA1I / Cav3.3), providing a candidate for the low-threshold calcium conductances recorded in HVC’s Area X-projecting neurons and interneurons [4, 130]. Relative to other T-type channels, CACNA1I channels are the most resistant to attenuation during high-frequency firing, exhibit current amplitude facilitation, and have more positive thresholds of activation and inactivation [88, 131, 132]. These properties might help to regulate excitability in HVC, which shows temporally-precise bursting during production of mature song [113]. CACNA1I is also an attractive candidate for development studies given that T-type calcium channels may underlie changes in intrinsic electrophysiological properties of HVC neurons during song learning [32].

CACNA1B, an N-type calcium channel, has a large single-channel conductance and is closely associated with the calcium-sensitive presynaptic vesicle release machinery [133–136]. The distinctive higher expression of CACNA1B in HVC and Area X suggests that these song nuclei are specialized for rapid and efficient neurotransmission. CACNA1E, an R-type calcium channel, functions in both dendritic calcium signaling and neurotransmitter release [35, 137]. In mammalian hippocampal neurons, these channels activate potassium channels within dendritic spines to limit depolarization in response to excitatory post-synaptic potentials [138, 139]. Additionally, CACNA1E^−/−^ mice are more resistant to seizure [140], possibly due to dampened excitability [141]. Depending on CACNA1E subcellular localization, lower expression in RA may translate to larger calcium transients in dendritic spines and/or reduce the potential for run-away excitability.

The modulatory α2δ (CACNA2D) and β (CACNB) subunits increase calcium currents as well as vesicle release probability [66, 142–145]. Their widespread high expression, particularly in the AFP, might thus relate to more efficient neurotransmission. The nearly identical expression patterns of α2δ and β in HVC and LMAN contrast with the divergent patterns of α1 subunits in these nuclei, suggesting that differences in calcium conductance might arise from unique combinations of α1 subunits rather than different auxiliary subunits. In turn, the higher expression of both modulatory α2δ subunits and pore-forming α1 subunits in Area X and HVC suggests that high levels of both these subunits are required. Finally, evidence that CACNA2D3 participates in neurexin-mediated retrograde signaling [146] suggests Area X might utilize a α2δ-dependent, inhibitory synaptic feedback mechanism that is unique among song nuclei.

Most γ (CACNG) subunits are transmembrane AMPA receptor (AMPAR) regulatory proteins, or TARPs, which modulate gating and synaptic targeting of AMPARs [147, 148]. A sparse population of Area X cells that resemble the large DLM-projecting neurons in Area X [12] showed enhanced expression of CACNG5, a TARP unique in its ability to increase AMPAR desensitization and deactivation rates [147]. Intriguingly, a population of sparse, large cells in Area X also expresses GluR4, the AMPAR subunit with the fastest kinetics [149], further suggesting that temporally precise signaling through AMPARs is particularly important for Area X projection neurons. In contrast, CACNG4 and CACNG8 are the most effective TARPs in slowing the desensitization, deactivation, and mEPSC decay rates of AMPARs [150]. The lower expression of CACNG4 in HVC, Area X, and LMAN and lack of a functional CACNG8 gene suggest that these nuclei rely on alternate mechanisms to elicit slow AMPA kinetics, such as differential splicing of AMPA subunits into their “flip” or “flop” forms [149]. CACNG7 selectively shuttles to the plasma membrane and boosts currents of calcium-permeable AMPARs while inhibiting membrane expression of calcium-impermeable ones [151, 152]. Thus, biasing AMPAR pools toward calcium permeability and enhancing calcium flow through AMPARs might be a shared specialization in RA, HVC, and Area X. In addition, CACNG7 can dampen CACNA1B (Cav2.2) expression in heterologous expression systems, but not in sympathetic neurons [153]. Accordingly, CACNA1B was not lower in any nuclei that showed higher CACNG7 expression. Finally, the non-TARP CACNG1 and CACNG6 decrease calcium currents by interacting with voltage-gated calcium channels. Birds lack CACNG6, but CACNG1 could potentially compensate for this gene loss.

The high expression of multiple intracellular calcium channels suggests that mobilization of internal calcium stores is important for HVC. While the role of TPCNs in neural processing is unknown, inositol 1,2,5-trisphosphate (IP3) receptors (ITPRs) and ryanodine receptors (RYRs) are implicated in plasticity, learning, and memory [154]. The higher expression of RYR2 and CACNA1C in Area X is also intriguing, given that RYR2 interacts with CACNA1C in muscle cells to coordinate excitation-contraction coupling [155]. Area X cells might thus funnel calcium ions through plasma membrane-bound CACNA1C to ER-bound RYR channels as a way to amplify calcium signaling. Lastly, ITPRs and RYRs do not overlap in location or direction of expression, suggesting the recruitment of distinct intracellular calcium stores and signaling pathways across song nuclei [156].

### Chloride channels

Of the gene families studied here, chloride channels are the least characterized and had a low incidence of differential expression. Considering their role in stabilizing the resting potential [36, 157], the higher expression of chloride channels in HVC and RA might help the membrane potential to reset despite large voltage fluctuations.

Passeriformes appear to lack CLCA genes. In mammals, these calcium-activated proteases are expressed predominantly in intestine and airway epithelium, and only a few are expressed in brain [78]. The absence of CLCAs suggests that songbirds rely on other mechanisms to regulate chloride transport in respiratory, digestive, and excretory tissues. Some CLCAs are expressed in olfactory sensory neurons (OSNs) in mammals, where they modulate chloride currents involved in olfactory signaling [158, 159]. Contrary to previous notions, birds use olfactory cues [160–162] and have large repertoires of olfactory receptor genes [42, 163–165], but downstream signal transduction mechanisms are unknown. In mammals, ~ 90% of the transduction current in OSNs is carried by the calcium-sensitive chloride channel ANO2 (TMEM16B) [166–168]. Because ANO2 is present in zebra finch and other avian species, birds appear to have the necessary machinery for olfactory transduction. Moreover, BEST2 - once the strongest candidate for transduction in OSNs [169] - is missing from zebra finch, consistent with the conclusion that BEST2 is not required for olfaction [170]. In sum, while the main carrier of olfactory transduction current (ANO2) may be conserved between mammals and birds, the lack of CLCAs suggests that regulation of this current may be markedly different in Passeriformes.

## Conclusions

We report here on the genomic identity and song system expression of three major ion channel families. The majority of these genes are conserved between birds and mammals, and the expression data paint a picture of abundant differential patterns, especially for sodium and calcium channel genes, in circuitry that supports the acquisition and production of learned vocalizations. We note that while changes in synaptic and intrinsic properties during the song learning period have been reported [11, 110, 111, 171], further studies of developmental regulation of ion channel genes in song nuclei are needed [172, 173]. Finally, this investigation establishes a foundation and framework for future studies to test the contribution of specific ion channel genes to distinct excitability properties of vocal nuclei in songbirds.

## Additional files

Additional file 1: **Table S1.** Documents synteny analysis, chicken orthologs, model completeness, sequence recovery data, and EST evidence for ion channel genes in the zebra finch genome. **Table S2.** details evidence supporting gene loss conclusions. (XLSX 29 kb)

Additional file 2: Overview of ortholog identification pipeline**.** Each box represents a step toward identifying an ortholog in zebra finch. Arrows connecting boxes indicate the most common workflows. Details of main pipeline (yellow) and all variations (Cases 1–6), including the use of zebra finch PacBio (pink) and other species (yellow), can be found in methods section. Dotted line indicates Case 4, where there is limited synteny information in taeGut1 and PacBio is required for verification (PDF 607 kb)

Additional file 3: Confirming orthology in the PacBio assembly using BLAST. Graphic summary of BLAST results demonstrating that SCN1B is present on gap-less PacBio scaffold MUGN01000920.1 with conserved exon structure and the conserved syntenic genes BCL3 and a fragment of RMB42 (see Fig. 2). Numbers indicate location (bases) along the scaffold. All alignments shown are non-avian RefSeqs. Double dashes indicate additional contracted sequence between selected regions shown. (PDF 234 kb)

Additional file 4: Quantitative analysis of zebra finch model completeness**.** A) Frequency histogram of genes by zebra finch/human model length ratio. B) Frequency histogram of genes by percent of post-recovery length. (PDF 183 kb)

Additional file 5: Visualization of sequence recovery for a gene with split models**.** The zebra finch gene, CACNA2D2, is displayed using the UCSC Genome Browser. A zoomed-in region from within the green rectangle is shown in the bottom panel to highlight the detailed structure of alignments. This gene has three partial zebra finch Ensembl models (dark red track) in a region with numerous gaps (black Gap tack). Note the dips in sequence quality scores (light blue track) surrounding the gaps. Alignment of a more complete chicken model (magenta arrowheads) reveals additional sequence blocks that are missing from the zebra finch model, displayed in the SeqRecovery BED track highlighted in yellow. Non-zebra finch RefSeqs (dark blue tracks) provide further support for blocks of additional sequence recovered through alignments of the chicken model. (PDF 336 kb)

Additional file 6: Sequence recovery BED track**.** A taeGut1 BED track of the sequence recovered from aligning chicken models. (TXT 19 kb)

Additional file 7: Expression of select ion channel genes in song nuclei**.** Representative in situ hybridization photomicrographs of select ion channel genes in song nuclei RA, HVC, Area X, and DLM. All genes are differentially expressed except for CACNA2D1 and CACNG5 in RA, which appear to label sparse populations of cells. Area X panels show select genes with enhanced expression in sparse populations of cells. Camera lucida drawings indicating the location of these nuclei can be found in Fig. 7a for RA, Fig. 8a for HVC, Fig. 9a for Area X, and Fig. 10a for DLM. Gene abbreviations are given in Table 1. All scale bars = 500 μm. (PDF 5215 kb)

## Data Availability

The datasets supporting the conclusions of this article are included within the article and Additional files 1, 2, 3, 4, 5, 6, 7. A GitHub repository for the sequence recovery analysis is available from [47]. Complete microarray datasets are available from [54] and details regarding curation methods can be found in [55]. High-resolution images of most of the in situ data presented here are publicly available on ZEBrA [50].

## Abbreviations

AFP: Anterior forebrain pathway
DLM: Medial nucleus of the dorsolateral thalamus
DM: Dorsomedial nucleus of the intercollicular complex
DMP: Direct motor pathway
HVC: Proper name
LMAN: Lateral magnocellular nucleus of the anterior nidopallium
NIf: Nucleus interfacialis of the nidopallium
nXIIts: Tracheosyringeal portion of the hypoglossal nerve nucleus
RA: Robust nucleus of the arcopallium
Uva: Thalamic nucleus uvaeformis
