# Supplementary figures and images for: Exploring the molecular basis of neuronal excitability in a vocal learner
Source: BMC Genomics. 2019 Aug 2;20:629. doi: 10.1186/s12864-019-5871-2 (PMC6679542; doi:10.1186/s12864-019-5871-2)

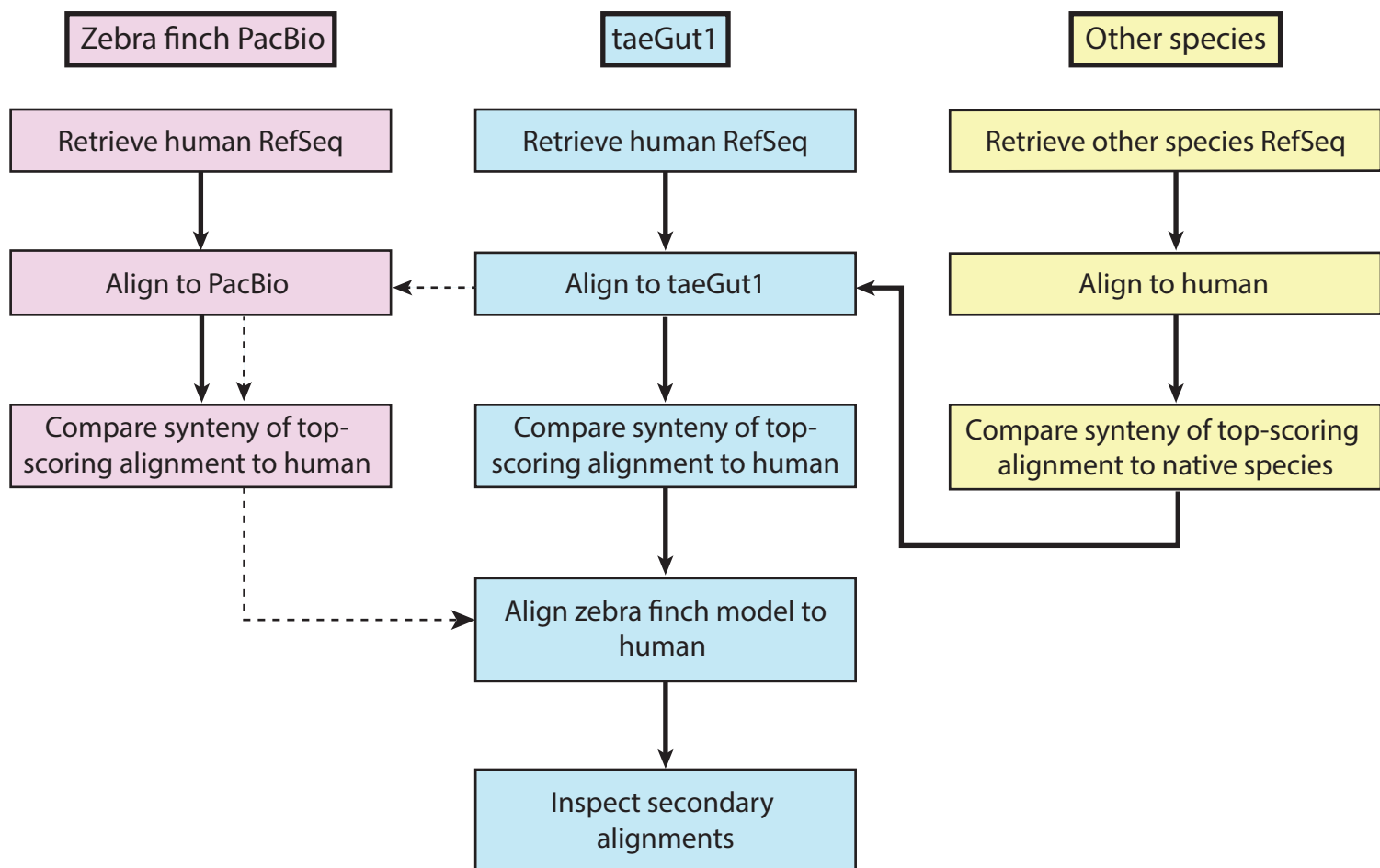

Supplement: Supplementary file 2 — Overview of ortholog identification pipeline. Each box represents a step toward identifying an ortholog in zebra finch. Arrows connecting boxes indicate the most common workflows. Details of main pipeline (yellow) and all variations (Cases 1–6), including the use of zebra finch PacBio (pink) and other species (yellow), can be found in methods section. Dotted line indicates Case 4, where there is limited synteny information in taeGut1 and PacBio is required for verification (PDF 607 kb) [file 12864_2019_5871_MOESM2_ESM.pdf]

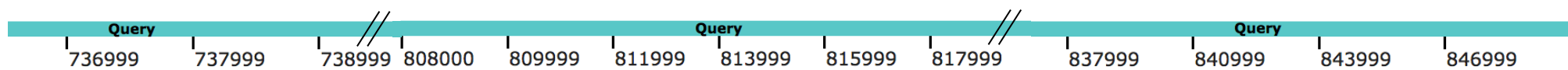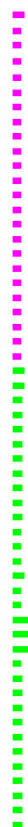

RBM42

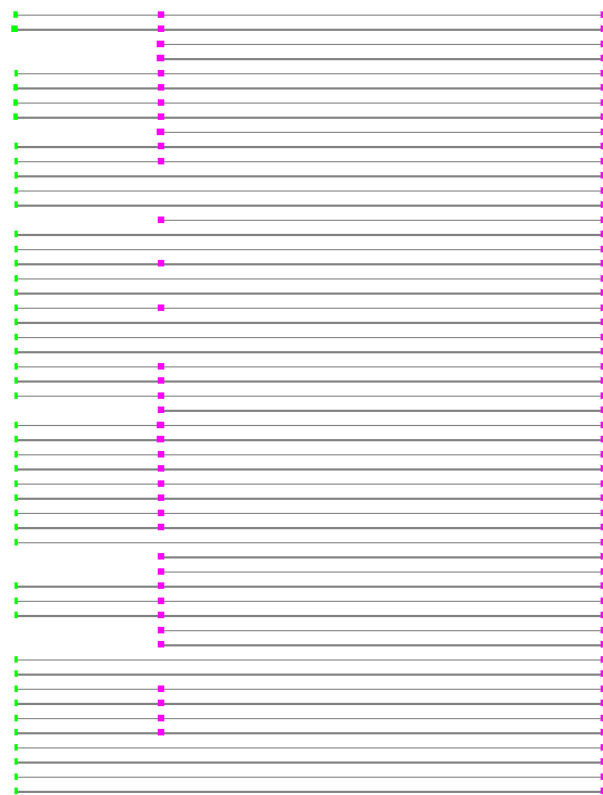

SCN1B

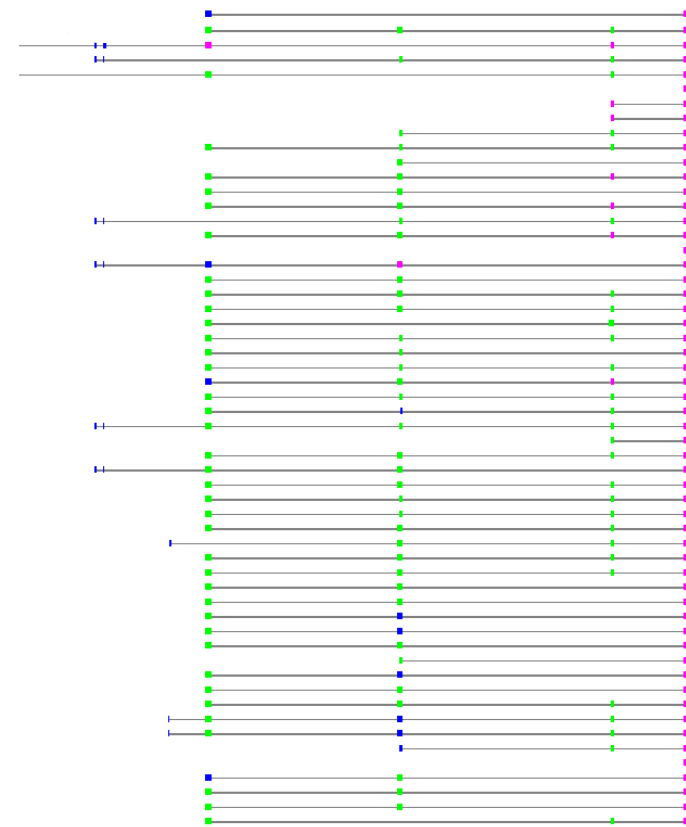

BCL3

Supplement: Supplementary file 3 — Confirming orthology in the PacBio assembly using BLAST. Graphic summary of BLAST results demonstrating that SCN1B is present on gap-less PacBio scaffold MUGN01000920.1 with conserved exon structure and the conserved syntenic genes BCL3 and a fragment of RMB42 (see Fig. 2). Numbers indicate location (bases) along the scaffold. All alignments shown are non-avian RefSeqs. Double dashes indicate additional contracted sequence between selected regions shown. (PDF 234 kb) [file 12864_2019_5871_MOESM3_ESM.pdf]

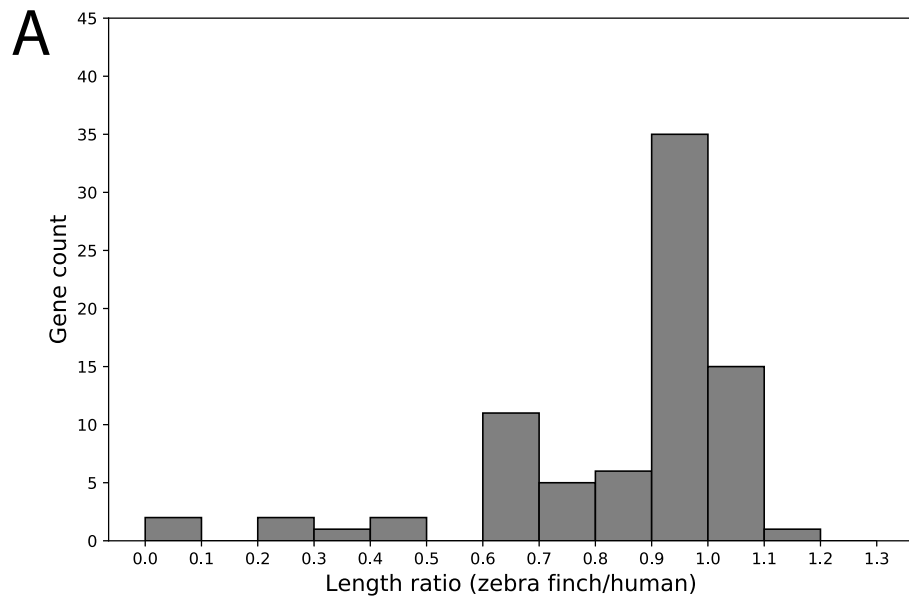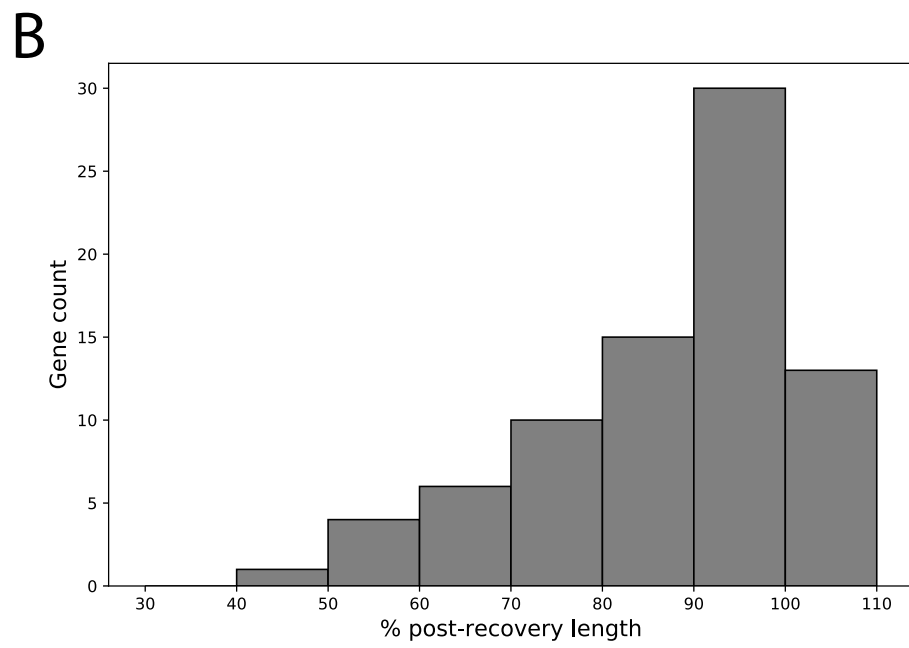

Supplement: Supplementary file 4 — Quantitative analysis of zebra finch model completeness. A) Frequency histogram of genes by zebra finch/human model length ratio. B) Frequency histogram of genes by percent of post-recovery length. (PDF 183 kb) [file 12864_2019_5871_MOESM4_ESM.pdf]

RA

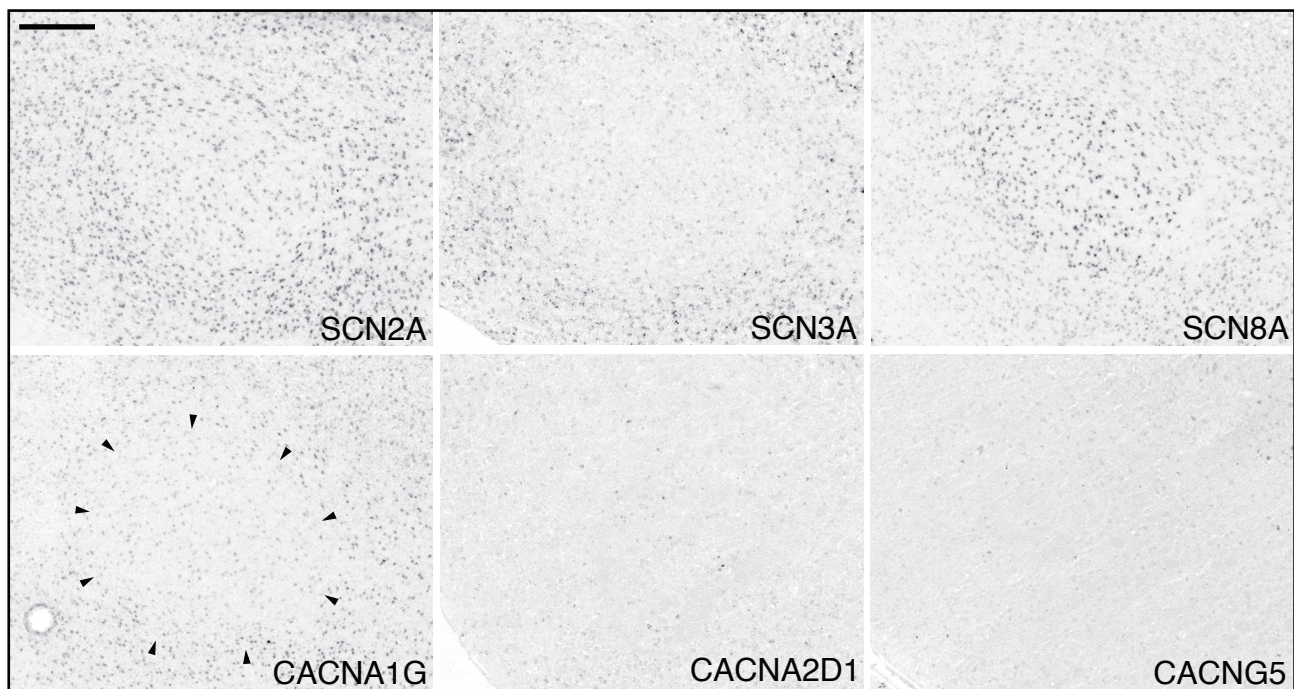

HVC

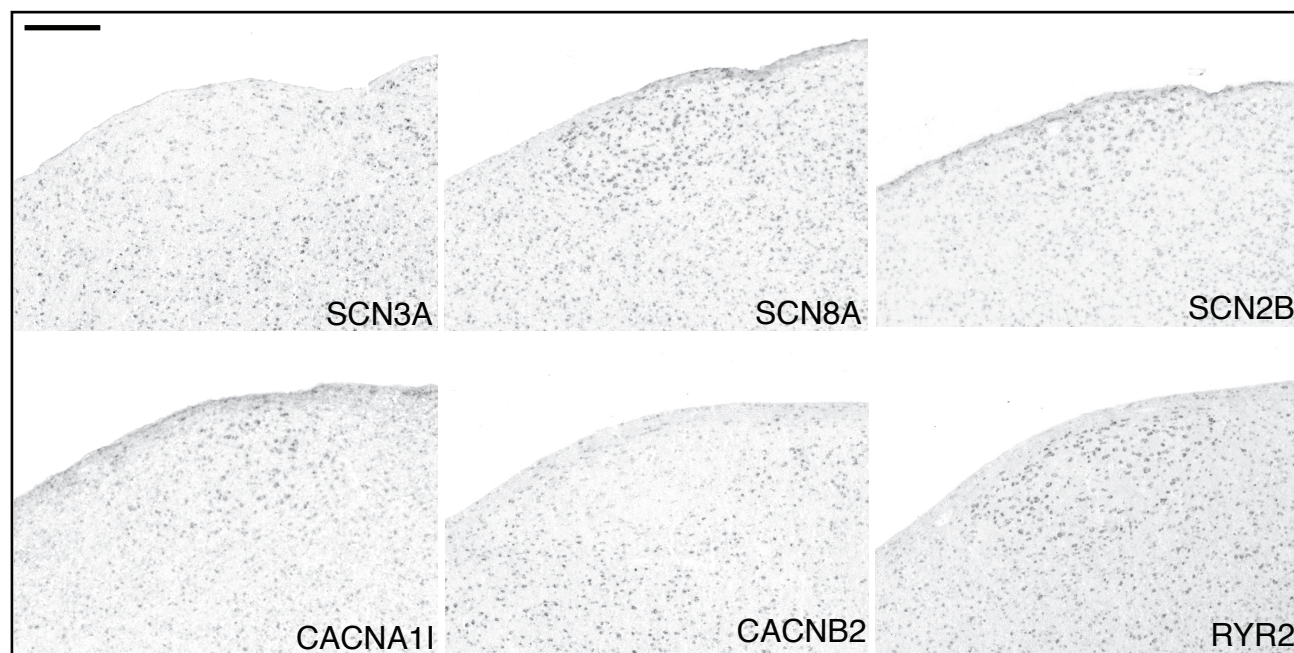

Area X

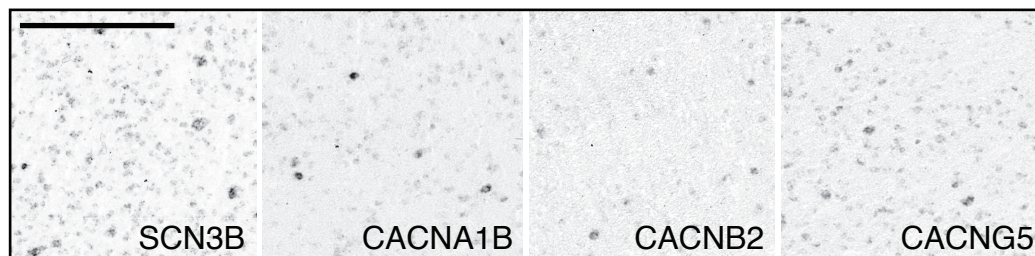

DLM

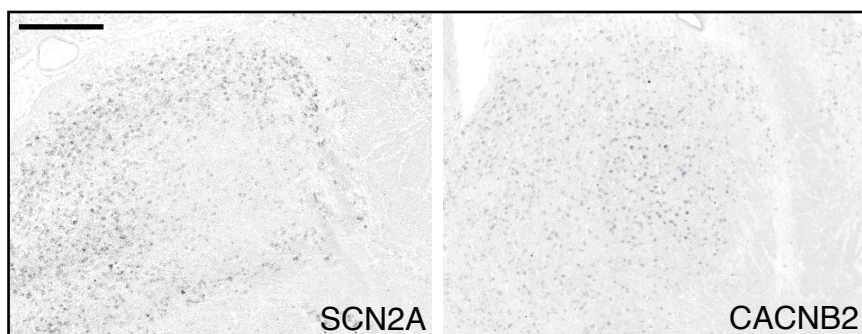

Supplement: Supplementary file 7 — Expression of select ion channel genes in song nuclei. Representative in situ hybridization photomicrographs of select ion channel genes in song nuclei RA, HVC, Area X, and DLM. All genes are differentially expressed except for CACNA2D1 and CACNG5 in RA, which appear to label sparse populations of cells. Area X panels show select genes with enhanced expression in sparse populations of cells. Camera lucida drawings indicating the location of these nuclei can be found in Fig. 7a for RA, Fig. 8a for HVC, Fig. 9a for Area X, and Fig. 10a for DLM. Gene abbreviations are given in Table 1. All scale bars = 500 μm. (PDF 5215 kb) [file 12864_2019_5871_MOESM7_ESM.pdf]
