# Supplementary material for: Exploring the molecular basis of neuronal excitability in a vocal learner
Source: BMC Genomics. 2019 Aug 2;20:629. doi: 10.1186/s12864-019-5871-2 (PMC6679542; doi:10.1186/s12864-019-5871-2)
Supplement: Supplementary file 5 — Visualization of sequence recovery for a gene with split models. The zebra finch gene, CACNA2D2, is displayed using the UCSC Genome Browser. A zoomed-in region from within the green rectangle is shown in the bottom panel to highlight the detailed structure of alignments. This gene has three partial zebra finch Ensembl models (dark red track) in a region with numerous gaps (black Gap tack). Note the dips in sequence quality scores (light blue track) surrounding the gaps. Alignment of a more complete chicken model (magenta arrowheads) reveals additional sequence blocks that are missing from the zebra finch model, displayed in the SeqRecovery BED track highlighted in yellow. Non-zebra finch RefSeqs (dark blue tracks) provide further support for blocks of additional sequence recovered through alignments of the chicken model. (PDF 336 kb) [file 12864_2019_5871_MOESM5_ESM.pdf]

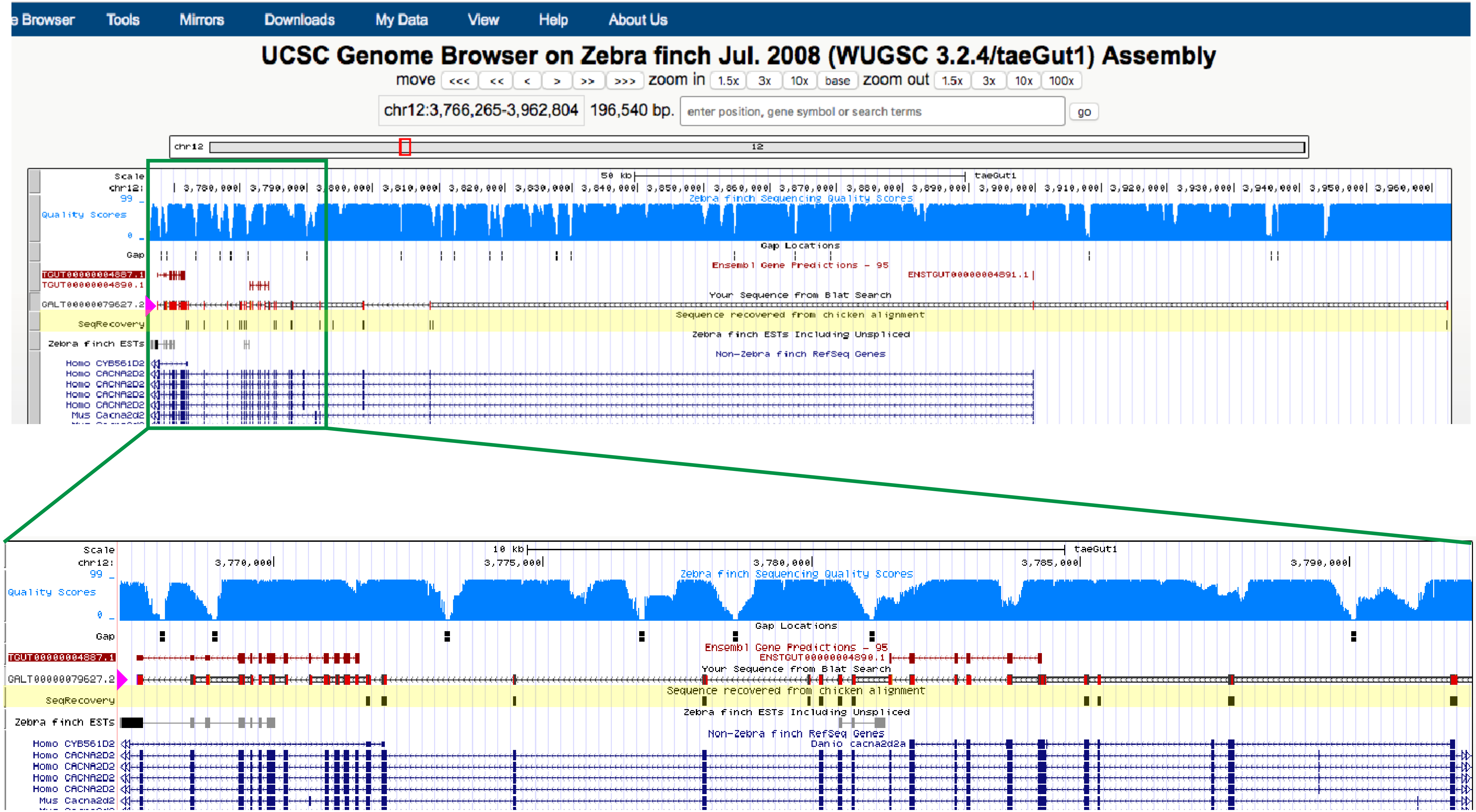
Browser
Tools
Mirrors
Downloads
My Data
View
Help
About Us
UCSC Genome Browser on Zebra finch Jul. 2008 (WUGSC 3.2.4/taeGut1) Assembly
move <<< << < > >> >>> zoom in 1.5x 3x 10x base zoom out 1.5x 3x 10x 100x
chr12:3,766,265-3,962,804 196,540 bp.
enter position, gene symbol or search terms
go
chr12
12
Scale
chr12:
50 kb
taeGut1
3,780,000
3,790,000
3,800,000
3,810,000
3,820,000
3,830,000
3,840,000
3,850,000
3,860,000
3,870,000
3,880,000
3,890,000
3,900,000
3,910,000
3,920,000
3,930,000
3,940,000
3,950,000
3,960,000
Zebra finch Sequencing Quality Scores
99
Quality Scores
0
Gap Locations
Gap
Ensembl Gene Predictions - 95
TGUT00000004887.1
TGUT00000004890.1
ENSTGUT00000004891.1
Your Sequence from Blat Search
GALT00000079627.2
Sequence recovered from chicken alignment
SeqRecovery
Zebra finch ESTs Including Unspliced
Zebra finch ESTs
Non-Zebra finch RefSeq Genes
Homo CYB561D2
Homo CACNA2D2
Homo CACNA2D2
Homo CACNA2D2
Homo CACNA2D2
Mus Cacna2d2
Scale
chr12:
10 kb
taeGut1
3,770,000
3,775,000
3,780,000
3,785,000
3,790,000
Zebra finch Sequencing Quality Scores
99
Quality Scores
0
Gap Locations
Gap
Ensembl Gene Predictions - 95
TGUT00000004887.1
ENSTGUT00000004890.1
Your Sequence from Blat Search
GALT00000079627.2
Sequence recovered from chicken alignment
SeqRecovery
Zebra finch ESTs Including Unspliced
Zebra finch ESTs
Non-Zebra finch RefSeq Genes
Danio cacna2d2a
Homo CYB561D2
Homo CACNA2D2
Homo CACNA2D2
Homo CACNA2D2
Homo CACNA2D2
Mus Cacna2d2
